# Evolutionary dynamics and biogeography of Musaceae reveal a correlation between the diversification of the banana family and the geological and climatic history of Southeast Asia

**DOI:** 10.1111/nph.13856

**Published:** 2016-02-01

**Authors:** Steven B. Janssens, Filip Vandelook, Edmond De Langhe, Brecht Verstraete, Erik Smets, Ines Vandenhouwe, Rony Swennen

**Affiliations:** ^1^ Botanic Garden MeiseNieuwelaan 38MeiseBE‐1860Belgium; ^2^ Laboratory of Tropical Crop ImprovementWillem de Croylaan 42LeuvenBE‐3001Belgium; ^3^ Plant Conservation and Population BiologyKU LeuvenKasteelpark Arenberg 31PO Box 2435LeuvenBE‐3001Belgium; ^4^ Naturalis Biodiversity CenterLeiden UniversityPO Box 9517Leiden2300RAthe Netherlands; ^5^ Bioversity InternationalWillem De Croylaan 42LeuvenBE‐3001Belgium; ^6^ International Institute of Tropical AgriculturePO Box 10, DulutiArushaTanzania

**Keywords:** ancestral area analysis, dispersal, diversification, *Ensete*, historical biogeography, *Musa*, *Musella*, Southeast Asia

## Abstract

- Tropical Southeast Asia, which harbors most of the Musaceae biodiversity, is one of the most species‐rich regions in the world. Its high degree of endemism is shaped by the region's tectonic and climatic history, with large differences between northern Indo‐Burma and the Malayan Archipelago. Here, we aim to find a link between the diversification and biogeography of Musaceae and geological history of the Southeast Asian subcontinent.
- The Musaceae family (including five *Ensete*, 45 *Musa* and one *Musella* species) was dated using a large phylogenetic framework encompassing 163 species from all Zingiberales families. Evolutionary patterns within Musaceae were inferred using ancestral area reconstruction and diversification rate analyses.
- All three Musaceae genera – *Ensete*,* Musa* and *Musella* – originated in northern Indo‐Burma during the early Eocene. *Musa* species dispersed from ‘northwest to southeast’ into Southeast Asia with only few back‐dispersals towards northern Indo‐Burma.
- Musaceae colonization events of the Malayan Archipelago subcontinent are clearly linked to the geological and climatic history of the region. *Musa* species were only able to colonize the region east of Wallace's line after the availability of emergent land from the late Miocene onwards.

## Introduction

Tropical Southeast Asia can be considered as one of the most biodiversity‐rich regions in the world. The region encompasses at least four biodiversity hotspots (Sundaland, Philippines, Wallacea and Indo‐Burma) and is renowned for its high plant species richness, harboring at least 10% of the world's endemic plants (Myers *et al*., 2000; Woodruff, 2010). Although the enormous plant biodiversity in Southeast Asia can be partly ascribed to its current geographic position in tropical and subtropical climatic zones, it is especially the region's complex tectonic and climatic evolution that has shaped the present species richness and high degree of endemism (Sodhi *et al*., 2004; Woodruff, 2010; Wong, 2011). When geographically and climatically comparing mainland Indo‐Burma – with its northern boundary situated in the tropical area of Yunnan (Southwest China) – with the islands of Sunda and Sahul, it is clear that their evolutionary history is remarkably different, despite being part of the same large Southeast Asian geographic region (de Bruyn *et al*., 2014; Deng *et al*., 2014). The northern area of tropical Southeast Asia (further referred to as northern Indo‐Burma), despite its high latitude and elevation, has a tropical moist climate. At present, this monsoon climate is maintained by mountain ranges in the north (e.g. Hengduan Mts) that keep out cold northern air during winter and by the presence of sufficient orographic rainfall and fog during the dry season. Plant fossils, sedimentary records and geochronology analyses have demonstrated that this warm and humid climate in the northern area of Southeast Asia has possibly been present since the late Oligocene–early Miocene (Yunnan Bureau of Geology and Mineral Resources, 1990). Therefore, it has been postulated that part of the Yunnan plateau (part of northern Indo‐Burma) has been covered with evergreen broad‐leaved forests throughout the last 25 million yr (Deng *et al*., 2014) and therefore has provided a relatively stable environment for tropical elements at such high latitude. By contrast, Malesia can be regarded as one of the earth's most geographically complex regions in the tropics (Thomas *et al*., 2011). Not only has the geography of the region been substantially altered since the early Eocene (*c*. 50 million yr ago, Ma), recent sea level fluctuations during the Plio‐ and Pleistocene have resulted in a continuous fusion and isolation of islands with mainland Sunda (Hall, 2001, 2002, 2009). Today, Malesia consists of over 20 000 islands, most of which are no larger than 10 km^2^, yet some, such as New Guinea, Sumatra and Borneo, belong to the largest islands on earth (Lohman *et al*., 2011). This island‐rich archipelago stands in strong contrast with the stable landmass that is found in the northern regions of Southeast Asia, and this most likely had a large impact on the evolution of the flora and fauna in the area.

Although the regional impact of the different tropical Southeast Asian subregions, such as Malesia (including Wallacea, Sundaland and Philippines) and northern Indo‐Burma (including Yunnan), on the overall floristic biodiversity has been confirmed (van Welzen & Slik, 2009; Zhu, 2013), it remains unclear to what extent past geological and climatic events in these subregions have affected the evolution of independent plant lineages. In this aspect, Musaceae is of particular interest. Musaceae is a small plant family that consists of three genera: *Ensete*,* Musa* and *Musella*. Whereas *Musella* is a monospecific genus native to southern Sichuan and northern Yunnan (Wu & Kress, 2000; Ma *et al*., 2011), *Ensete* comprises eight species and is distributed in Madagascar, tropical Africa and Asia (Champion, 1967; Väre & Häkkinen, 2008). The majority of Musaceae species, however, belong to the genus *Musa*, which has a distribution range that coincides roughly with the different tropical Southeast Asian hotspots (Sundaland, Philippines, Wallacea and Indo‐Burma). From a taxonomic point of view, *Musa* is regarded as a problematic group as a result of past and present typification issues (Häkkinen & Väre, 2008), the struggle of collecting quality herbarium material because of the large, fleshy habit (Argent, 2000) and the frequent presence of ephemeral flowers (Liu *et al*., 2002; Chiu *et al*., 2011). As a result, the number of species in the genus *Musa* is estimated to be between 60 and 70. Members of Musaceae are characteristic elements of tropical and subtropical forests, where they often occur as ecologically important understory species. Although there are notable exceptions, most *Ensete* and *Musa* species cannot endure persistent drought or cold. Compared with the other wild Musaceae lineages, *Musella* has a rather extreme ecological preference, being able to tolerate much drier and colder habitats. The rather limited ecological niche that is occupied by Musaceae species, in combination with its mainly Southeast Asian distribution (except for the African *Ensete* species), provides an interesting tool to detect biogeographic patterns at the level of tropical forest regions in Southeast Asia. In addition, the slightly moderate dispersal capabilities, mainly by frugivore vertebrates (Medellín & Gaona, 1999; Tang *et al*., 2007; Fleming & Kress, 2013), and the seemingly limited gene flow through seeds (Ge *et al*., 2005; Burgos‐Hernández *et al*., 2013) contribute to the unraveling of biogeographic events within a broad evolutionary time scale.

This article examines the origin and evolution of the Musaceae family with special emphasis on the tempo and timing of diversification of the genus *Musa* in relation to its distribution range throughout tropical Southeast Asia. Evolutionary relationships and age estimates within major Musaceae clades are inferred to gain more insight into the complex processes of speciation and extinction that shaped the current banana biodiversity in tropical Southeast Asia. In addition, we discuss to what extent past climatic and geological events, such as the continuous sea level fluctuations during the Pleistocene and the collision of the Sunda and Sahul Shelf, influenced the radiation of *Musa* in tropical Southeast Asia.

## Materials and Methods

### Taxon sampling

The taxon sampling represents five *Ensete* species (six accessions), 38 *Musa* species (63 accessions) and one species (one accession) of *Musella* (Supporting Information Table S1). In order to correctly estimate node ages for *Ensete*,* Musa* and *Musella*, we extended the Musaceae dataset with 156 Zingiberales species and two outgroup taxa (Methods S1).

### Molecular protocols and sequence analyses

DNA was isolated using a modified cetyltrimethylammonium bromide (CTAB) protocol. In total, four gene markers (*rps16*,* atpB*‐*rbcL*,* trnL‐F* and internal transcribed spacer, ITS) were amplified and sequenced under standardized protocols. Aligned sequence data matrices were analyzed using Bayesian inference methodology (Methods S2).

### Divergence time analysis

Age estimates within the Musaceae family were inferred using a large‐scale dating method in which the whole Zingiberales is covered. Such an approach allows us to incorporate multiple fossil calibration points and consequently reduces the bias of a single calibration point. In addition, because of the still uncertain position of Musaceae as one of the earlier diversified branches within the Zingiberales order (e.g. Kress *et al*., 2001; Barrett *et al*., 2013; Yockteng *et al*., 2013), it was difficult to confidently assign a suitable sister lineage that could support a calibration point at the root. As a result, we opted to extend the Musaceae sampling with representatives of all other families within the Zingiberales (see earlier) to obtain a robust framework for node age estimation. Well‐identified fossils that belong to representatives of the order Zingiberales were used to calibrate the topology: fossil seeds of *Ensete oregonense* found in middle Eocene deposits in Oregon (USA), used as a minimum age constraint of 43 Ma for the crown node of *Ensete* and *Musella* (= stem node of *Ensete*) (Manchester & Kress, 1993); leaves of *Zingiberopsis attenuata* from the Paleocene Paskapoo Formation of Alberta (Canada), applied as a minimum age of 65 Ma for the crown node of the Zingiberaceae family (Hickey & Peterson, 1978). A third calibration point was set to calibrate the root of the topology. For this, we used the previously computed age estimate for the Zingiberales of Magallon & Castillo (2009) to calibrate the crown node age of the order at 87 Ma, which corresponds to the dating analysis of Bell *et al*. (2010). This age estimate also matches rather well with the oldest known fossil of the Zingiberales – *Spirematospermum chandlerae* – which is estimated to have a minimum age of 83.5 Ma (Friis, 1988). The two fossil calibration points used in this study were assumed to follow a log‐normal distribution with an offset that equals the age of the fossil calibration point, a mean of 1.0 and a standard deviation of 1.0, resulting in 43.4–62.3 and 65.4–84.3 Ma 95% intervals for calibration points 1 and 2, respectively. The third calibration point was given a normal distribution with a mean value of 87 and standard deviation of 2.0 (83.1–90.9 Ma 95% interval).

The molecular clock hypothesis was tested using a chi‐square likelihood ratio test (Felsenstein, 1988) and demonstrated that the substitution rates in the combined dataset are not clock‐like (*P *<* *0.001 for all markers). Beast v.1.8.0 (Drummond & Rambaut, 2007) was used to compute divergence times (Methods S3).

### Ancestral area reconstruction

Ancestral areas within Musaceae were reconstructed using the dispersal–extinction–cladogenesis (DEC) model as implemented in Lagrange (Ree *et al*., 2005; Ree & Smith, 2008). The maximum clade credibility tree obtained from the Beast dating analysis was chosen as input tree. Based on Takhtajan's (1986) floristic regions of the world and Olson's terrestrial ecoregions of the world (Olson *et al*., 2001), we delimited 12 geographic regions encompassing the current distribution of *Ensete*,* Musa* and *Musella* (A, Africa; B, Southeast India and Sri Lanka; C, northern Indo‐Burma; D, South China; E, southern Indo‐Burma; F, Sumatra and Malayan Peninsula; G, Borneo; H, Philippines; I, New Guinea and surrounding islands; J, Northwest Australia; K, Lesser Sunda Islands; L, Sulawesi). Coordinates and elevation ranges from the Global Biodiversity Information Facility (GBIF) were used to determine to which geographic region *Musa* species sampled in the current article belong. GBIF data were generated with the rgbif‐package (Chamberlain *et al*., 2013) as implemented in R, and screened for erroneous localities before distribution range assessment. Distribution ranges were additionally checked against the literature to determine which *Musa* species have a putative enlarged distribution range as a result of human dispersal since the Holocene epoch, *c*. 10 000 Ma (e.g. *Musa balbisiana*; Simmonds, 1959). In order to infer the impact of evolutionary events that shaped the current banana biodiversity, only non‐human‐induced distribution ranges are taken into account in this study. The maximum range size was defined at three. The dispersal effectiveness was set at symmetric with the possibility to change in time by defining time slices. These time frames were added because parts of Malesia did not exist at the time of origin of *Musa* and *Ensete*–*Musella*. In total, four time slices were defined based on the study of Hall (2002, 2009): 0–10 Ma (Pleistocene to late Miocene), 10–20 Ma (late to early Miocene), 20–30 Ma (early Miocene to Oligocene) and 30–55 Ma (Oligocene to early Eocene). Dispersal rates between the different geographic areas for each of the time slices are shown in Table S2.

### Musaceae diversification

Semi‐logarithmic lineage through time (LTT) plots were generated for both *Musa* and *Ensete*–*Musella* to assess the timing and tempo of speciation and to visualize the net diversification for both genera (LTT plots compensate for incomplete taxon sampling). The LTT plot obtained was examined to evaluate whether shifts in diversification rate had occurred during the evolutionary history of the two largest clades in Musaceae: *Musa* and *Ensete*–*Musella*. We chose to combine the latter genera for the diversification analyses as *Musella* is the sister genus of *Ensete* and consists of only one species. In order to avoid putative inflation of the speciation rate caused by the inclusion of more than one specimen per species, we decided to only include one individual per (sub‐)species. One thousand randomly selected ‘post‐burnin’ chronograms from the posterior distribution of the Beast dating analyses were used to assess credibility envelopes that indicate LTT plot variation amongst both lineages. LTT plots were generated with the ape package (Paradis *et al*., 2004) as implemented in R. The impact of an incomplete taxon sampling on the LTT pattern was tested by generating simulated phylogenies that cope with a proportion of missing data using Phylogen v.1.1 (Rambaut, 2002). Simulated phylograms were generated under a Yule process with branch lengths of the simulated trees for *Musa* and *Ensete*–*Musella* rescaled to 37.9 and 44.7 Ma, respectively, using TreeEdit 1.0 (Rambaut & Charleston, 2002).

Two different methods were used to test diversification rates within *Musa* and *Ensete*–*Musella*. For the first method, diversification analyses were conducted with Bayesrate v.1.6.3 (Silvestro *et al*., 2011). This software package is able to estimate speciation rates in a Bayesian framework under different diversification models (e.g. pure birth, birth–death). In addition, Bayesrate copes with uncertainties in node divergence and incomplete taxon sampling (see later). As input for the diversification analyses, 1000 randomly generated trees from the posterior Beast distribution were used for a Markov Chain Monte Carlo (MCMC) run of 100 000 iterations, sampled every 100^th^ generation. Bayes factors (interpretation follows Kass & Raftery, 1995) were applied to assess the best‐fit model of lineage diversification. Net diversification rates were calculated for *Musa* and *Ensete*–*Musella* under a Yule process with rate shifts (Rabosky, 2006), assuming a minimum of two rate shifts based on the LTT plots obtained.

The second method applied to infer shifts in net diversification rate within Musaceae was Bayesian analysis of macroevolutionary mixtures (BAMM) 1.0 (Rabosky, 2014). BAMM is a Bayesian‐based approach that implements reversible jump MCMC to account for different diversification models and rate variation through time. The maximum clade credibility tree derived from the Beast analysis was used as input data. Four MCMC simulations were run for 1 million generations sampled each 1000^th^ generation. In addition, sampling fractions were taken into account for both *Ensete*–*Musella* and *Musa* (see later). Tracer was used to examine for chain convergence and to check whether the effective sample size (ESS) exceeded 200. Diversification rate plots were obtained via the R packages BAMMtools 2.0.2 (Rabosky *et al*., 2014) and ape (Paradis *et al*., 2004), as implemented in R.

In order to compensate for an incomplete taxon sampling in both Bayesrate and BAMM, it is important to assess the exact number of Musaceae species. However, as a result of the problematic *Musa* taxonomy, it is challenging to infer the exact number of species within the genus. Based on the study of Häkkinen & Väre (2008) and the number of newly described *Musa* species since 2008 (e.g. Chen *et al*., 2014; Dey *et al*., 2014), we estimate the number of *Musa* species at *c*. 70 distinct entities. In addition, based on the rather clear delimitation of most *Musa acuminata* subspecies in our phylogenetic analyses and the general, often arbitrary, delineation of natural lineages in species or subspecies (Hamilton & Reichard, 1992; Mayr, 2000), we decided to consider the different subspecies of *Musa acuminata* (and likewise also the subspecies of other *Musa* species) as different entities for both diversification rate methods. As a result, the clade‐specific sampling proportion of *Musa* was fixed at 0.54. For *Ensete* and *Musella*, the total number of species is currently estimated at nine (Väre & Häkkinen, 2008). However, because of the different phylogenetic positions of both *E. glaucum* individuals sampled in our study, we considered them as two distinct entities. Therefore, the clade‐specific sampling proportion of *Ensete*–*Musella* was set at 0.66.

### Biogeographic diversification patterns within tropical Southeast Asia

In order to investigate the regional impact of different tropical Southeast Asian subregions, diversification rates were compared between *Musa* species that were native to either the climatically and geologically stable biodiversity hotspot northern Indo‐Burma or to the more geologically variable Malesia (including Sundaland, Philippines and Wallacea biodiversity hotspots). Using a Bayesian implementation of the binary‐state speciation and extinction model (BiSSE‐BMA) implemented in Bayesrate v.1.6.3 (Silvestro *et al*., 2011), we were able to compute the fit of distinct diversification models using thermodynamic integration for marginal likelihood estimation. In the case of *Musa*, the BiSSE algorithm provides the most appropriate model to infer diversification rates, as no species occur in both northern Indo‐Burma and Malesia, and thus a binary model could be applied.

The best‐fit model for the BiSSE analysis was chosen from eight models with different constraint settings and degrees of freedom that were tested in a run of 100 000 iterations for 10 categories each. Computed Bayes factors were interpreted following Kass & Raftery (1995). Parameters for the best model were then applied in an MCMC run of 1000 randomly generated trees from the posterior Beast distribution with 100 000 iterations on each tree, sampled every 1000^th^ generation. Based on inferred distribution ranges from GBIF and the literature (see the Ancestral area reconstruction subsection), *Musa* species that occurred in northern Indo‐Burma were scored as 0, whereas those on Malesia were scored as 1. Similar to the earlier diversification analyses, we considered subspecies as distinct taxonomic units. In total, the analysis contained 17 northern Indo‐Burmese representatives and 27 Malesian representatives (Table S1). Five species distributed in southern Indo‐Burma remained unassigned. Distribution range data of all currently recognized *Musa* species were used to infer proportions of missing data for the two groups, resulting in a clade‐specific sampling proportion of the Indo‐Burmese group at 0.51 and a clade‐specific sampling proportion of the Malesian group at 0.65.

## Results

### Sequence characteristics and phylogenetic results

Sequence characteristics for all data matrices analyzed are summarized in Table S3. Despite the inability to sequence all loci, their absence did not influence the overall phylogenetic results, as sufficient nucleotide variation was present throughout.

**Figure 1:**
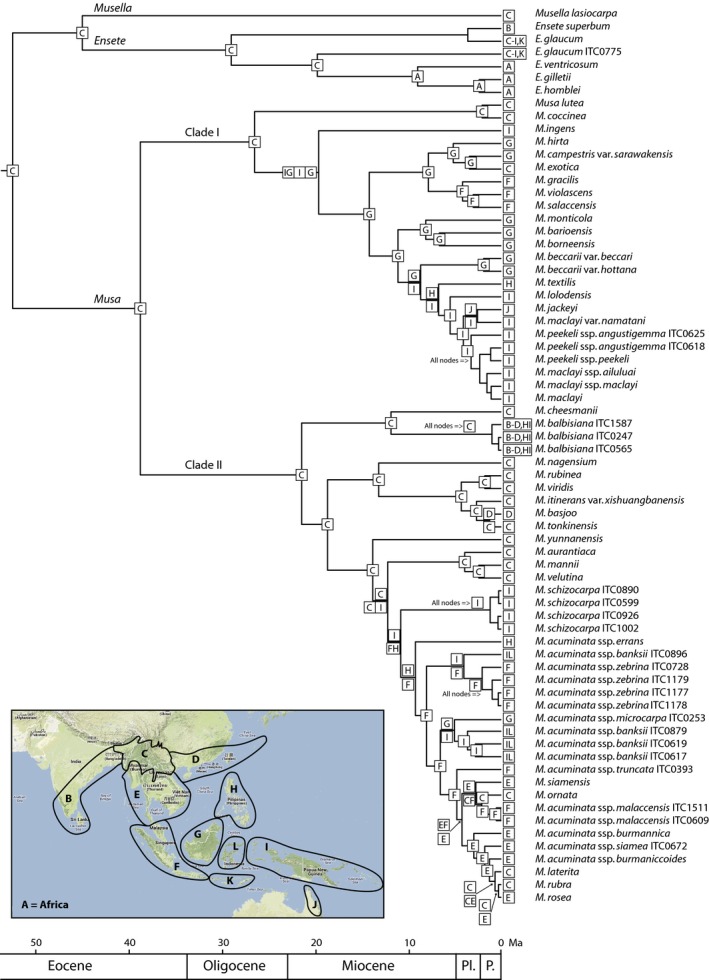
Maximum clade credibility tree of Musaceae (detail of the large Zingiberales phylogram in Supporting Information Fig. S1). Ancestral area reconstructions (AARs) with the highest likelihood values are shown as boxes at each node. A single box refers to a specific distribution range, whereas multiple boxes depicted either above or below the branches indicate alternative AARs. In the case of low likelihood values, more than one ancestral area is shown. The following abbreviations are used: A, Africa; B, Southwest India and Sri Lanka; C, northern Indo‐Burma; D, South China; E, southern Indo‐Burma; F, Sumatra and Malayan Peninsula; G, Borneo; H, Philippines; I, New Guinea and surrounding islands; J, Northwest Australia; K, Lesser Sunda Islands; L, Sulawesi; Pl., Pliocene; P., Pleistocene. Lagrange provides two ranges per node; in cases in which only one range is present at a certain node, both lineages inherited the same ancestral distribution range. ‘All nodes =>’ above a branch indicates that all nodes of this clade are characterized by the same ancestral area.

At high phylogenetic level within Zingiberales, Bayesian topologies of the plastid (*trnL‐F*,* rps16* and *atpB‐rbcL*) and nuclear (ITS) datasets did not show incongruent clades and, except for the unresolved position of the Marantaceae family in the plastid dataset, Zingiberales interfamilial relationships were identical for both datasets (Fig. S1). Although the interfamilial relationships were, on average, moderate to well resolved for both datasets, the combined phylogeny showed better resolution and generally higher support values. Hence, we used the combined Bayesian phylogeny for further discussion. Musaceae is a highly supported monophyletic lineage (Bayesian posterior probability (BPP), 0.99) and is sister to the remainder of Zingiberales, a result that is in accordance with Kress *et al*. (2001), Wikström *et al*. (2001), Bell *et al*. (2010) and Yockteng *et al*. (2013). Within the remainder of Zingiberales families, the lineage towards Heliconiaceae, Strelitziaceae and Lowiaceae (BPP, 0.97) is sister with high support (BPP, 0.98) to the clade consisting of Zingiberaceae, Costaceae, Marantaceae and Cannaceae (BPP, 0.97). In addition, Strelitziaceae and Lowiaceae form a clade (BPP, 0.88) sister to Heliconiaceae, whereas Costaceae is sister to Zingiberaceae (BPP, 0.87) and Marantaceae is sister to Cannaceae (BPP, 0.98). Intergeneric relationships within each Zingiberales family (except for Musaceae) are not discussed further in depth, as this is not the focus of the study. For Musaceae, both plastid and nuclear topologies are generally highly congruent. Nevertheless, within the lineage towards the *Musa* and *Rhodochlamys* sections, the positions of the *M. acuminata* ssp. *schizocarpa* clade, *M. balbisiana* clade and *M. acuminata* ssp. *siamea* differ between the two phylogenetic trees, yet these incongruences are only weakly supported (Fig. S2). The incongruency test was conducted on the pruned Zingiberales dataset in which only Musaceae representatives were kept. The partition homogeneity test did not demonstrate incongruency between plastid and nuclear datasets (*P *=* *0.06). For the combined data matrix, Bayesian inference yielded a well‐resolved topology with moderate to strong support for the majority of the lineages. The combined topology is used for further discussion throughout the text. Musaceae forms a well‐supported clade that is split into two major lineages: *Musa* (BPP, 0.98) and *Ensete*–*Musella* (BPP, 0.99). Within the *Ensete*–*Musella* sister relationship, the latter genus is well supported (BPP, 0.98). The genus *Musa* is divided into two large clades. One clade (Clade I; BPP, 0.99) is well resolved and consists only of taxa that belong to the sections *Ingentimusa*,* Australimusa* and *Callimusa* (Fig. 1). The other large *Musa* clade (Clade II; BPP, 0.98) is characterized by less resolved relationships and contains representatives of sections *Musa* and *Rhodochlamys* (Fig. 1).

### Divergence time estimates

Within 30 million generations, stasis of the different chains as well as ESS values over 200 were obtained. The Beast maximum clade credibility tree is depicted in Fig. S3. The divergence time for Musaceae was estimated at 51.9 Ma (61.2–45.6 Ma 95% highest priority density (HPD)), suggesting an early Eocene origin (Figs 1, S3). Likewise, the split between sister genera *Ensete* and *Musella* is also situated in the early Eocene at 44.7 (48.2–43.1 Ma 95% HPD; calibration point used is 43 Ma). The initial radiation of *Ensete* occurred in the Oligocene at a mean estimated age of 28.5 Ma (42.1–16.9 Ma 95% HPD). The diversification of *Musa* started during the late Eocene (mean age estimate, 37.9 Ma; 50.5–24.5 Ma 95% HPD). Clade I in the genus *Musa* containing species of sections *Ingentimusa*,* Australimusa* and *Callimusa* diversified in the Oligocene at *c*. 26.3 Ma (38.9–16.0 Ma 95% HPD), whereas Clade II (represented by species of sections *Musa* and *Rhodochlamys*) started to radiate *c*. 6 million yr later in the early Miocene (mean age estimate, 20.9 Ma; 30.4–13.3 Ma 95% HPD).

### Ancestral area reconstruction

Hardly any unambiguous ancestral area splitting was observed (Fig. 1). Relative probability values for the nodes of interest were nearly always at least 25% higher than the next ancestral area alternative. Of the 73 nodes analyzed, 61 had a relative probability value of > 50% for one of the ancestral areas, whereas 33 nodes had a relative probability value above 90%. The current ancestral area analysis not only revealed an origin in the northern Indo‐Burmese (C) hotspot for the Musaceae family, but also showed that the genera *Musella*,* Musa* and *Ensete* all originated in northern Indo‐Burma (Fig. 1). Within *Ensete*, a single colonization event from northern Indo‐Burma gave rise to the African species of the genus. At the time of divergence of *Musa* Clade I (containing species of sections *Ingentimusa*,* Australimusa* and *Callimusa*), a split can be observed into a northern Indo‐Burmese (C) and a Bornean (G) lineage. Species from Clade I, which are found on New Guinea and the surrounding islands (I) or Malayan Peninsula/Sumatra (F), are derived from a Bornean ancestor, whereas species occurring in northern Australia (J) and the Philippines (H) have a New Guinean (including surrounding islands) (I) ancestry (Fig. 1). Within Clade II, the earliest diversified lineages have an Indo‐Burmese origin, whereas the more recently diversified lineages that occur elsewhere on Malesia have an ancestor situated in the region of Sumatra and the Malayan Peninsula (Fig. 1).

### Musaceae diversification

**Figure 2:**
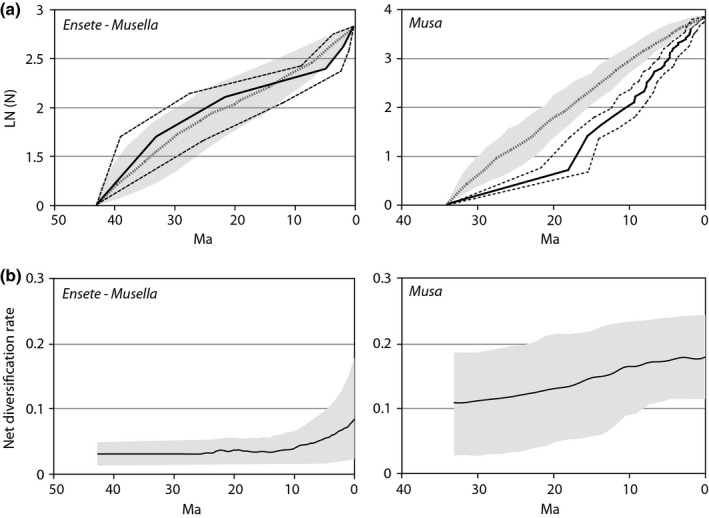
Diversification plots. (a) Semi‐logarithmic lineage through time (LTT) plots of *Ensete*–*Musella* and *Musa*. The solid black lines represent the mean LTT plots of the empirical analysis, and the dashed black lines represent the upper and lower boundaries of the 95% confidence intervals. Simulated LTT plots (under pure birth) with 95% confidence intervals are indicated in gray (mean value represented by dashed gray line). (b) Net diversification rates through time under a Yule process are shown as solid black lines. 95% highest posterior density intervals are indicated in gray. Ma, million years ago.

The LTT plots of *Ensete*–*Musella* and *Musa* reveal a different evolutionary history for the two major lineages within Musaceae (Fig. 2a). The LTT pattern (including 95% confidence intervals) of *Ensete*–*Musella* demonstrates a good fit between the observed patterns and those predicted under a constant diversification model. By contrast, the *Musa* LTT plot indicates an initially low diversification rate until 17 Ma, followed by an episode of increased diversification.

**Figure 3:**
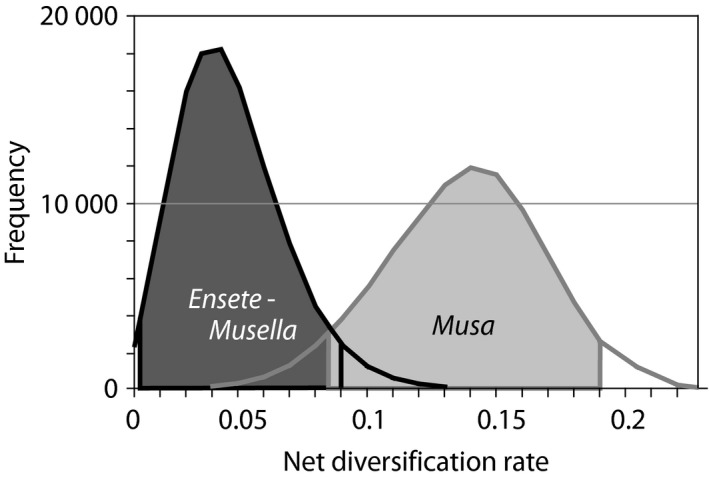
Overall net diversification of *Ensete*–*Musella* and *Musa* as calculated with Bayesrate. Ninety‐five percent highest posterior density for *Ensete*–*Musella* and *Musa* are depicted in dark gray and light gray, respectively.

These results are corroborated by the diversification rate through time analyses obtained with Bayesrate (Fig. 2b), in which the net diversification rate for *Musa* gradually increased from 0.11 species Ma^−1^ to 0.18 species Ma^−1^. For the *Ensete*–*Musella* clade, the net diversification rate remained constant at 0.037 species Ma^−1^ until the last 5 Ma, when an increase towards 0.08 species Ma^−1^ was observed (Fig. 2b). When comparing overall extinction and speciation rates between the two lineages, it becomes clear that the genus *Musa* has a high mean speciation rate of 0.15 species Ma^−1^ (95% HPD, 0.19–0.52 species Ma^−1^; Fig. 3), whereas the *Ensete*–*Musella* lineage has a low mean speciation rate of only 0.04 species Ma^−1^ (95% HPD, 0.02–0.17 species Ma^−1^; Fig. 3).

**Figure 4:**
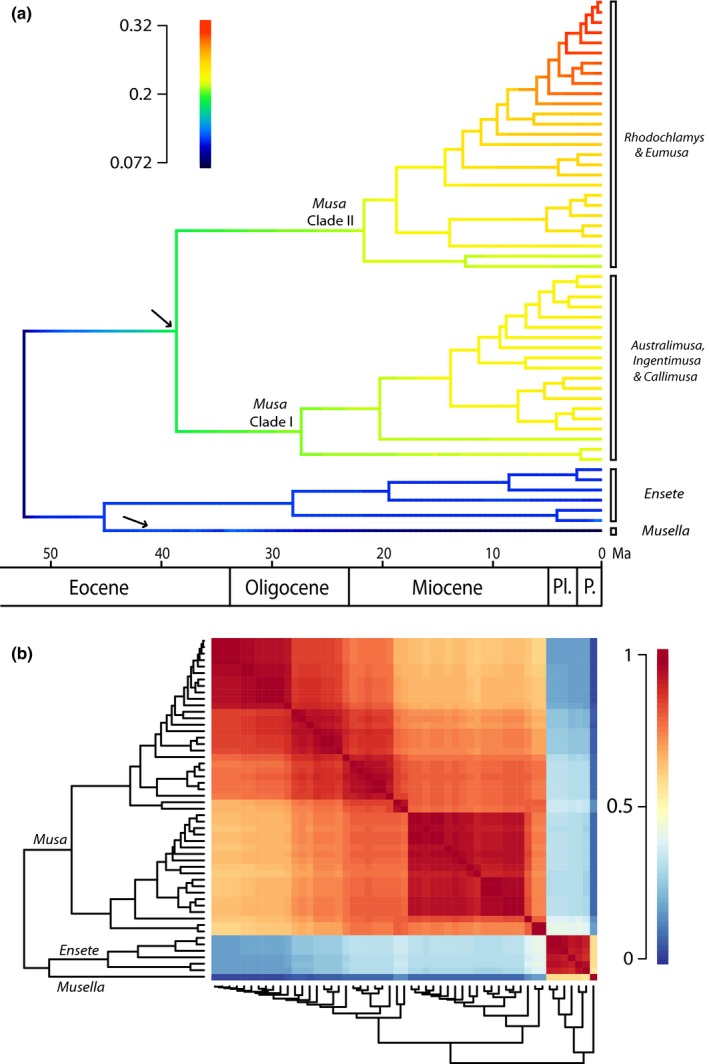
Diversification patterns in Musaceae. (a) Phylorate plot with estimates of net diversification rates of the Musaceae phylogenetic tree obtained from the Bayesian analysis of macroevolutionary mixtures (BAMM) analyses. Colors at each time along the branches indicate net diversification rates. Arrows indicate shifts in diversification rate based on shifts in marginal probability along the phylogram. (b) Macroevolutionary cohort matrix for speciation in Musaceae. Every cell in the matrix is coded by a color indicating the pairwise probability between two lineages and their common macroevolutionary rate regime. Dark red indicates a pairwise probability of 1, whereas a deep blue color marks a pairwise probability of shared macroevolutionary dynamics of 0. In total, three major cohorts could be identified representing a different rate regime for each Musaceae genus. In addition, within each genus, rate dynamics are highly correlated.

Shifts in diversification rate in BAMM were not assessed on a set of generated trees from the posterior Beast distribution, but on the Beast maximum clade credibility tree. BAMM MCMC likelihood reached convergence with ESS exceeding 200. Maximum shift credibility revealed two diversification shifts: one in *Musella* and one at the initial diversification of *Musa* (Fig. 4a). These shifts were confirmed by a cohort analysis conducted with BAMMtools (Fig. 4b).

### Biogeographic diversification patterns within tropical Southeast Asia

**Figure 5:**
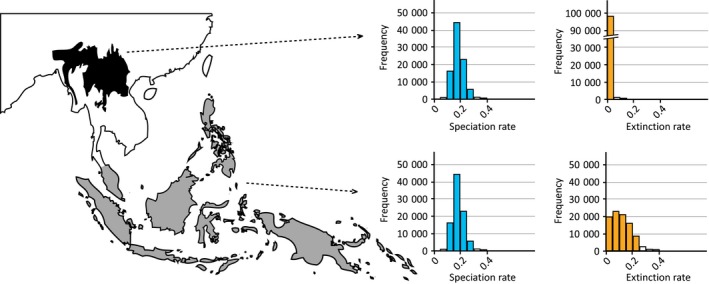
Posterior estimates of speciation and extinction rates, in blue and orange, respectively, associated with the area of distribution of *Musa* (northern Indo‐Burma vs Malesia). Speciation rates were defined a priori to be equal (Supporting Information Table S4). White bars fall out of the 95% confidence interval. The northern Indo‐Burmese region is indicated in black, whereas Malesia is indicated in gray.

Diversification analyses showed that the presence of *Musa* species in one of the two distinct tropical Southeast Asian subregions (northern Indo‐Burma vs Malesia) is correlated with changes in extinction and speciation rate within that genus. Of the eight models tested, the BiSSE‐BMA analysis in Bayesrate found the model with equal speciation rates, equal transition rates and different extinction rates (pure birth for northern Indo‐Burmese species and birth–death for Malesian species) as a best fit for the data (Table S4). In addition, this model differs significantly from the other models tested (Table S4). Speciation rates for both regions appear to be similar (*λ*_0_ = *λ*_1_ = 0.20; CI [95% confidence interval] = 0.12–0.26), as are state transition rates (*q*_0_ = *q*_1_ = 0.14; CI = 0.08–0.2; Fig. 5). However, the analysis revealed that extinction rates are significantly higher in Malesia than in the northern Indo‐Burmese subregion. *Musa* evolution in northern Indo‐Burma is seemingly correlated with a low extinction rate (*μ*_1_ = 0.001; CI = 0–0.02), in contrast with the evolutionary patterns on Malesia, where the extinction rate is higher (*μ*_0_ = 0.13; CI = 0.06–0.21).

## Discussion

### Phylogenetic relationships and dating

The phylogenetic relationships obtained within Musaceae are largely in congruence with the results of Liu *et al*. (2002), Wong *et al*. (2002), Nwakanma *et al*. (2003), Li *et al*. (2010) and Christelová *et al*. (2011). Musaceae forms a monophyletic clade in which the currently recognized genera (*Musa*,* Ensete* and *Musella*) form well‐delimited monophyletic lineages (Figs 1, S3). Within the genus *Musa*, two well‐supported main clades are delineated. Whereas the first clade (Clade I) contains species from sections *Ingentimusa*,* Australimusa* and *Callimusa*, its sister lineage consists only of *Eumusa* and *Rhodochlamys* species (Clade II). Among the most recently diverged lineages in both Clade I and Clade II, there appears to be significantly lower resolution and/or support (Figs 1, S3). Although this is most likely the result of a lack of sufficient nucleotide variation within the overall sequence data matrix, traces of sequence incongruence between different closely related lineages is probably also the reason for these mainly unresolved branches. Recently, Hribova *et al*. (2011) have found that, within Musaceae, ITS is prone to frequent polymorphism. This ITS polymorphism could be the result of inter‐ or intragenomic duplication events, as well as interspecific hybridization (Hribova *et al*., 2011), and could explain the resolution problems within some Musaceae lineages. Although several species for which more than one specimen is sampled form well‐supported monophyletic lineages (e.g. *M. acuminata* ssp. *zebrina*,* M. acuminata* ssp. *schizocarpa*,* M. balbisiana*), accessions of *M*. *acuminata* ssp. *banksii* are scattered throughout the large *Musa acuminata s.l*. group. This inconsistency at subspecies level could be an indication of either putative hybrid origin and subsequent introgression or of distinct evolutionary lineages that show large morphological resemblance.

Present dating analysis of Musaceae (and Zingiberales) revealed age estimates that are slightly younger than those found by Janssen & Bremer (2004) and Christelová *et al*. (2011). Compared with the study of Christelová *et al*. (2011), the currently obtained crown node ages of Musaceae (51.9 Ma, early Eocene) and *Musa* (37.9 Ma, late Eocene) are *c*. 17 and 13 Ma younger, respectively. In fact, our estimated crown node age of Musaceae and stem node ages of *Musa*,* Ensete* and *Musella* are in accordance with the study of Kress & Specht (2005, 2006). Nevertheless, our Musaceae stem node age of 88.5 Ma (= Zingiberales crown node age) does not correspond to the 96.5–110 Ma age estimation of Kress & Specht (2005, 2006) for the same node. Furthermore, the timing of initial diversification set at *c*. 26.3 Ma for the *Ingentimusa*–*Australimusa*–*Callimusa* clade and 20.9 Ma for the *Eumusa*–*Rhodochlamys* clade corresponds well with the results of Christelová *et al*. (2011). This demonstrates that, despite the differences in sampling strategy, calibration point selection and gene marker selection, similar results are obtained when compared with earlier attempts to date Musaceae and its genera (Kress & Specht, 2005, 2006; Christelová *et al*., 2011).

### Dispersal events and evolutionary history of *Musella*

The banana family originated and diversified during the early Eocene in northern Indo‐Burma. In a time span of < 10 Ma after the origin of the family, all three Musaceae genera (*Musa*,* Ensete* and *Musella*) were established within the northern Indo‐Burmese region. Each of these three genera is characterized by a different evolutionary history.

The monospecific genus *Musella* is a typical element of Southwest China at the northern border of Indo‐Burma. In contrast with the other Musaceae genera, *Musella* apparently did not radiate or disperse into Southeast Asia. Instead, it is adapted to a different ecological niche compared with its close relatives as it can also grow and reproduce in drier and colder habitats (Liu *et al*., 2002). *Musella* used to be widespread in the subtropical evergreen Yunnan Plateau forests (Olson *et al*., 2001; Liu *et al*., 2002) until the suitable habitat was fragmented as a result of extensive cultivation (Ma *et al*., 2011). In addition to the narrow distribution range in Southeast Asia and the unique ecological preference within Musaceae, *Musella lasiocarpa* populations have a low inter‐population genetic distance (Pan *et al*., 2007). Interestingly, the lineage towards *Musella* has been subjected to a significant negative shift in diversification rate compared with its sister genus *Ensete* (Fig. 4).

### Dispersal events and evolutionary history of *Ensete*

Initial radiation within the genus *Ensete* occurred within the northern Indo‐Burmese region during the Oligocene. Colonization events towards South India (*E. superbum*; Western Ghats) and Africa (*E. gilletii*,* E. homblei* and *E. ventricosum*; tropical Africa) occurred very recently during the Pliocene and Miocene, respectively. The dispersal towards Africa could be the result of a single long‐distance dispersal event from northern Indo‐Burma or of a gradual overland dispersal via an Arabian corridor during a more mesic period. Although both hypotheses are possible, the latter is probably more likely. *Ensete* seeds are large and heavy (5–18 mm; Lane, 1955), and usually do not spread far as new seedlings are often found close to the mother plant (Baker & Simmonds, 1953). Moreover, *E. ventricosum* and *E. gilletii* have a rather continuous nondisjunct distribution range in tropical Africa that favours a more gradual dispersal pattern rather than a sudden long‐distance dispersal event. Also, the presence of Eocene *Ensete* fossils in North America points towards an ancient overland dispersal event, which probably followed the Boreotropical migration route present during warmer periods of the Eocene epoch. However, in addition to monkeys, birds are also considered to act as dispersal agents for *Ensete* (Baker & Simmonds, 1953), which could provide evidence in favour of a long‐distance dispersal event.

The overall net diversification rate of *Ensete* (as analyzed together with *Musella*) is significantly lower than that of *Musa*, and is comparable with the primary diversification rate of the banana family, as both *Musa* and *Musella* are characterized by a significant shift in diversification rate (Fig. 4). Moreover, there is no indication that the genus was subjected to a high degree of extinction, implying that the overall speciation rate was seemingly low throughout the evolution of *Ensete*. However, diversification rate analyses through time indicate a slight increase in diversification rate since the Pliocene. The low overall diversification rate could be explained by the possibility of the occupation of a broader ecological niche by *Ensete* representatives, their steady dispersal capacity and the presence of self‐pollinating flowers (Champion, 1967). For example, *Ensete* contains the most widespread species within Musaceae, such as *E. ventricosum* (East to Central Africa), *E. gilletii* (Central to West Africa) and *E. glaucum* (distributed in India, Indo‐Burma and Malesia), and also fossils of the genus have been found on the North American continent (*E. oregonense*; Manchester & Kress, 1993; Manchester, 1994).

### Dispersal events and evolutionary history of *Musa*

**Figure 6:**
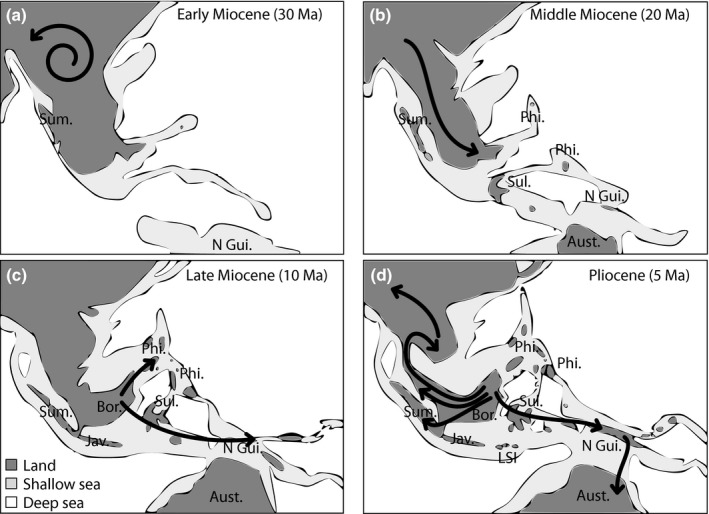
Tertiary maps of Southeast Asian paleogeography and land availability. (a) Early Miocene (30 million yr ago, Ma). (b) Middle Miocene (20 Ma). (c) Late Miocene (10 Ma). (d) Pliocene (5 Ma). Dark black lines show the dispersal patterns of *Musa* along Southeast Asia reflecting a qualitative and generalized interpretation of Fig. 2. The arrow indicates the direction of dispersal. Spiral arrow indicates *in situ* diversification. Aust., Australia; Bor., Borneo; Jav., Java; LSI, Lesser Sunda Islands; N Gui., New Guinea; Phi., Philippines; Sul., Sulawesi; Sum., Sumatra.

The earliest diversification of *Musa* occurred in northern Indo‐Burma during the late Eocene. In total, two main colonization events from northern Indo‐Burma towards the rest of Southeast Asia (including Malesia) took place, each followed by a substantial localized diversification. Within Clade I (including *Ingentimusa*,* Callimusa* and *Australimusa*), the ancestral lineages started to leave the northern Indo‐Burmese region during the Oligocene, whereas the first dispersal of Clade II (including *Eumusa* and *Rhodochlamys*) from the region (followed by a known speciation event) only took place during the late Miocene. Based on the current results, it is likely that dispersal from northern Indo‐Burma towards Malesia occurred over land (Figs 1, 6). From the Miocene onwards, forest‐covered land connections from continental Asia towards the Sunda Shelf were present for longer periods (Hall, 2001, 2002, 2009; Morley, 1999). In addition, during episodes of low sea level during the Pleistocene, lowland rainforests covered parts of the currently flooded Sunda Shelf between Borneo, Sumatra, Java and the Malayan Peninsula (Morley, 1999). For Clade II, dispersal into Malesia from northern Indo‐Burma took place via the Malayan Peninsula (and Sumatra), whereas, for Clade I, colonization of Malesia occurred mostly via Borneo. After northern Indo‐Burma, Borneo is the most species‐rich region in tropical Southeast Asia in terms of *Musa* diversity. Interestingly, Borneo's *Musa* diversity is situated in the lineage towards *Ingentimusa*,* Australimusa* and *Callimusa* (Clade I) and not in the lineage towards *Eumusa* and *Rhodochlamys* (Clade II), as the only Bornean member of *Eumusa* (*M. acuminata* ssp. *microcarpa*) is probably the result of a recent colonization from the Malayan Peninsula/Sumatra. Since the early Miocene, Borneo has accumulated *Musa* representatives by both the accretion of immigrants and *in situ* diversification (Figs 1, 6). Moreover, Borneo served as a center of secondary radiation for the *Ingentimusa*–*Australimusa*–*Callimusa* lineage, as can be observed by the intense within‐area diversification. From here, several dispersal events have taken place to Papua, Sulawesi and the Philippines since the late Miocene–Pliocene. For the *Eumusa*–*Rhodochlamys* clade, not Borneo, but the Malayan Peninsula (and Sumatra), played an important role in the diversification and dispersal of this clade during the Miocene. These late colonization events towards central and eastern Malesia for both *Eumusa*–*Rhodochlamys* and *Ingentimusa*–*Australimusa*–*Callimusa* are linked to the geological history of the region. Whereas significant parts of western Malesia were already present during the Tertiary, most land east of the Wallace line only appeared during the late Miocene and Pliocene when the Sahul Shelf gradually collided with the Sunda Shelf (Figs 1, 6). Only after the availability of the emergent land from the late Miocene did *Musa* species colonize the region east of Wallace's line several times. This ‘west to east’ dispersal trend in Southeast Asia is in accordance with recent biogeographic studies on other plant taxa in the region (e.g. *Aglaia*, Muellner *et al*., 2008; *Pseuduvaria*, Su & Saunders, 2009; *Begonia*, Thomas *et al*., 2011).

Although there is a general trend in *Musa* to colonize the Southeast Asian subcontinent from northwest to southeast, a few recently originated lineages have redispersed to mainland Southeast Asia from the south. Recolonization of northern Indo‐Burma (*M. ornata*,* M. laterita* and *M. exotica*) took place from the Malayan Peninsula/Sumatra via southern Indo‐Burma (Figs 1, 6). Interestingly, all lineages that are distributed in southern Indo‐Burma are not of northern Indo‐Burmese ancestry, but have a recent Malesian origin dating back to the Pliocene (Figs 1, 6). Therefore, it is reasonable to assume that seasonal forests or a savannah‐like habitat, in which *Musa* representatives could not be maintained, once covered considerable parts of southern Indo‐Burma. This assumption is supported by paleoclimatic reconstruction, fossil pollen records, geomorphological data and vegetation modeling analyses suggesting the existence of a dry seasonal climate corridor across the Sunda Shelf from Java towards the Malay–Thai Peninsula during the Pleistocene (Bird *et al*., 2005; Cannon *et al*., 2009; Wurster *et al*., 2010).

### Geographic impact on the diversification of *Musa*

The present data are in accordance with the results of de Bruyn *et al*. (2014), in which Indo‐Burma and Borneo are considered to be major evolutionary hotspots for Southeast Asian biodiversity. In addition, our results show that, within the Indo‐Burmese region, a large distinction can be made between northern and southern Indo‐Burma for *Musa*. The contrast in *Musa* diversity between northern and southern Indo‐Burma is probably associated with the drastic impact of recent climate fluctuations (see earlier) on the southern part of Indo‐Burma, not being able to maintain evergreen rainforest – and thus suitable habitat for bananas – during periods of increased drought (Bird *et al*., 2005; Cannon *et al*., 2009; Wurster *et al*., 2010). Also, a large distinction in biota formation can be observed between the major evolutionary hotspots, that is northern Indo‐Burma and Borneo (embedded in Malesia) (de Bruyn *et al*., 2014; Merckx *et al*., 2015). Geological and climatic processes associated with the onset of the early Miocene collision (Fig. 6; Hall, 2001, 2002, 2009), together with the recent Plio‐ and Pleistocene gradual cooling and accompanying climatic fluctuations (Wurster *et al*., 2010), shaped the current biodiversity in both regions (Lohman *et al*., 2011). Whereas the northern Indo‐Burmese region was probably characterized by a rather steady relatively warm and humid climate from the late Oligocene that could support broad‐leaved evergreen vegetation (Deng *et al*., 2014), Malesia experienced frequent habitat contraction and expansion caused by sea level fluctuations and different regional responses to cyclic climatic changes. In addition, most of the current Malesian landmass only rose above sea level from the early Miocene onwards as a result of the collision of the Sunda and the Sahul Shelf (Hall, 2001, 2009; Lohman *et al*., 2011; de Bruyn *et al*., 2014). Therefore, despite the presence of some putative rainforest refugia on Malesia (e.g. Borneo), most of the region has been subject to considerable habitat changes since the Miocene (Morley, 1999; Cannon *et al*., 2009). This geological and climatic difference between northern Indo‐Burma and Malesia is also reflected in the diversification of *Musa* species that occur in these regions. The probabilistic framework used in this study revealed unexpected asymmetries between speciation and extinction rates among species from northern Indo‐Burma and Malesia that could be associated with the different geographic regions in which they occur (Fig. 5).

Species that are distributed in the northern Indo‐Burmese region are associated with a lower extinction rate compared with those that are present on Malesia. Speciation rates appear to be similar between species of both regions, although an increased speciation rate for the Malesian *Musa* species was expected because of increased connection–disconnection events related to sea level fluctuations (de Bruyn *et al*., 2014). The low extinction rate for northern Indo‐Burmese *Musa* species corresponds well with the stable warm and humid climate that has occurred in northern Southeast Asia since the late Oligocene–early Miocene, and in which evergreen broad‐leaved forests have probably been continuously present (Yunnan Bureau of Geology and Mineral Resources, 1990; Deng *et al*., 2014). The increased extinction rate of the Malesian *Musa* species might be associated with the periodic Pleistocene inundation of the Sunda Shelf and the subsequent loss of suitable habitat. Moreover, the region has been substantially exposed to habitat changes since the Miocene, resulting in frequent shifts from evergreen tropical rainforest to drier habitats that were unsuitable for *Musa* representatives (Cannon *et al*., 2009; de Bruyn *et al*., 2014).

## Author contributions

S.B.J. and R.S. planned and designed the research. S.B.J., F.V. and B.V. analyzed the data. I.V. provided material. S.B.J., R.S., E.D.L., F.V., E.S. and B.V. wrote the manuscript.

## Supporting information

Please note: Wiley Blackwell are not responsible for the content or functionality of any supporting information supplied by the authors. Any queries (other than missing material) should be directed to the *New Phytologist* Central Office.

**Fig. S1** Beast maximum clade credibility tree of Zingiberales.
**Fig. S2** Bayesian consensus phylogram of plastid and nuclear dataset.
**Fig. S3** Beast phylogram of the combined plastid and nuclear dataset of Musaceae.
**Table S1** Accession numbers, voucher data and origin of plant material for taxa included in the combined DNA analyses of Zingiberales–Musaceae
**Table S2** Dispersal probabilities between the different Southeast Asian (and African) areas
**Table S3** Sequence characteristics
**Table S4** Model fit of area‐dependent diversification
**Methods S1** Taxon sampling.
**Methods S2** Molecular protocols and sequence analyses.
**Methods S3** Beast detail methods, parameters and settings.
