# Supplementary material for: Evolutionary dynamics and biogeography of Musaceae reveal a correlation between the diversification of the banana family and the geological and climatic history of Southeast Asia
Source: New Phytol. 2016 Feb 1;210(4):1453–65. doi: 10.1111/nph.13856 (PMC5066818; doi:10.1111/nph.13856)
Supplement: Supplementary file 1 — Fig. S1 Beast maximum clade credibility tree of Zingiberales. Fig. S2 Bayesian consensus phylogram of plastid and nuclear dataset. Fig. S3 Beast phylogram of the combined plastid and nuclear dataset of Musaceae. Table S1 Accession numbers, voucher data and origin of plant material for taxa included in the combined DNA analyses of Zingiberales–Musaceae Table S2 Dispersal probabilities between the different Southeast Asian (and African) areas Table S3 Sequence characteristics Table S4 Model fit of area‐dependent diversification Methods S1 Taxon sampling. Methods S2 Molecular protocols and sequence analyses. Methods S3 Beast detail methods, parameters and settings. [file NPH-210-1453-s001.pdf]

New Phytologist

New Phytologist Supporting Information Figs S1–S4, Tables S1–S3 and Methods S1–S3

Article title: Evolutionary dynamics and biogeography of Musaceae reveal a correlation between the diversification of the banana family and the geological and climatic history of Southeast Asia

Authors: Steven B. Janssens, Filip Vandelook, Edmond De Langhe, Brecht Verstraete, Erik Smets, Ines Vandenhouwe and Rony Swennen

Article acceptance date: 7 December 2015

The following Supporting Information is available for this article:

Fig. S1 BEAST Maximum clade credibility tree of Zingiberales.

Fig. S2 Bayesian consensus phylogram of plastid and nuclear dataset.

Fig. S3 BEAST phylogram of the combined plastid and nuclear dataset of Musaceae.

Table S1 Accession numbers, voucher data and origin of plant material for taxa included in the combined DNA analyses of Zingiberales-Musaceae

Table S2 Dispersal probabilities between the different Southeast Asian (and African) areas

Table S3 Sequence characteristics

Table S4 Model fit of area-dependent diversification

Methods S1 Taxon sampling.

Methods S2 Molecular protocols and sequence analyses.

Methods S3 BEAST detail methods, parameters and settings.

Table S1 Accession numbers, voucher data and origin of plant material for taxa included in the combined DNA analyses of Zingiberales-Musaceae. *Musa* taxa marked with a circle, asterisk or dash represent species that were used to infer diversification patterns. °, northern Indo-Burmese species; *, Malesian species; -, southern Indo-Burmese or East Asian species.

Family	Species	Voucher	*atpB-rbcL*	ITS	*rps16*	*trnL-F*
**Cannaceae**	*Canna indica*	19981130-03 (BR)	KU215245	KU215028	KU214891	KU215151
	Canna paniculata	-	-	AY673069	AY656159	AY140423
**Costaceae**	*Chamaecostus cuspidatus*	19381338 (BR)	-	KU215041	-	KU215165
	Chamaecostus subsessilis	-	-	AY994717	KJ011420	AY994555
	Cheilocostus speciosus	-	-	KC878573	-	AY994557
	Costus allenii	19730144 (BR)	KU215248	KU215031	KU214894	KU215154
	Costus amazonicus	-	-	AY041032	KJ011354	AY994586
	Costus arabicus	19730142 (BR)	KU215251	KU215034	KU214897	KU215156
	Costus barbatus	-	-	AY041031	KJ011357	AY994585
	Costus chartaceus	-	-	AY972911	KJ011360	AY994559
	Costus claviger	-	-	AY972882	KJ011361	AY994584
	Costus deistelii	19615174 (BR)	KU215255	KU215038	KU214901	-
	Costus dinklagei	-	-	AY994750	KJ011366	AY994596
	Costus dubius	19594350 (BR)	KU215257	KU215040	KU214903	KU215161
	Costus erythrocoryne	-	-	AY972886	-	AY994579
	Costus erythrophyllus	19730145 (BR)	KU215247	KU215030	KU214893	KU215153
	Costus gabonensis	-	-	AY994747	KJ011371	AY994593
	Costus guanaiensis	-	-	AY972883	KJ011374	AY994577
	Costus laevis	-	-	AY041035	KJ011377	AY994575
	Costus lateriflorus	-	-	AY994734	KJ011379	AY994574
	Costus letestui	-	-	AY972939	KJ011380	AY994573
	Costus lucanusianus	19620189 (BR)	KU215256	KU215039	KU214902	KU215160
	Costus maculatus	-	-	AY994731	-	AY994571
	Costus malortieanus	10005061 (BR)	KU215246	KU215029	KU214892	KU215152
	Costus montanus	19726433 (BR)	KU215249	KU215032	KU214895	-
	Costus mosaicus	-	-	AY994728	-	AY994568
	Costus phaeotrichus	-	-	AY994721	KJ011396	AY994561
	Costus pictus	10005272 (BR)	KU215250	KU215033	KU214896	KU215155
	Costus plicatus	-	-	AY041030	KJ011400	AY994565
	Costus pulverulentus	-	-	AY041029	AY656160	AY994563
	Costus scaber	19812689 (BR)	KU215252	KU215035	KU214898	KU215157
	Costus spectabilis	-	-	AY994718	KJ011406	AY994556
	Costus stenophyllus	-	-	AY994720	KJ011408	AY994560
	Costus talbotii	-	-	AY994716	KJ011412	AY994554
	Costus tappenbeckianus	-	-	AY994715	-	AY994553
	Costus vargasii	19860011 (BR)	KU215254	KU215037	KU214900	KU215159
	Costus varzearum	-	-	AY994714	KJ011413	AY994551

	Costus villosissimus	-	-	AY994713	KJ011414	AY994550
	Costus zingiberoides	19860010 (BR)	KU215253	KU215036	KU214899	KU215158
	Dimerocostus strobilaceus	19726435 (BR)	KU215258	-	KU214904	KU215162
	Monocostus uniflora	19750179 (BR)	KU215259	-	KU214905	KU215163
	Tapeinochilos ananasae	19610260 (BR)	KU215260	-	KU214906	KU215164
**Heliconiaceae**	*Heliconia acuminata*	19830761 (BR)	KU215261	-	-	KU215166
	Heliconia angusta	19750486 (BR)	-	-	KU214909	-
	Heliconia caribaea	-	FJ428018	FJ428106	FJ428109	FJ428179
	Heliconia densiflora	19880067 (BR)	KU215263	-	-	-
	Heliconia hirsuta	Van Caeckenberghe s.n. (BR)	KU215267	-	KU214912	-
	Heliconia humilis	19861386 (BR)	KU215265	-	KU214910	KU215168
	Heliconia illustris	10005273 (BR)	KU215262	-	KU214907	-
	Heliconia indica	19730253 (BR)	KU215264	-	KU214908	KU215167
	Heliconia psittacorum	-	FJ428016	FJ428105	FJ428108	FJ428180
	Heliconia rickardiana	19940052-53 (BR)	KU215268	-	KU214913	-
	Heliconia rostrata	19822412 (BR)	KU215266	KU215042	KU214907	KU215169
**Lowiaceae**	*Orchidantha chinensis*	-	FJ428061	FJ428181	FJ428153	FJ428181
	Orchidantha fimbriata	-	-	AF434879	AF430098	FJ621300
	Orchidantha maxillarioides	19074031 (BR)	KU215269	-	KU214914	KU215170
	Orchidantha siamensis	-	-	AF434887	AF430106	AF431622
**Marantaceae**	*Afrocalathea rhizantha*	-	-	EU605908	EF382847	EU647816
	Calathea altissima	-	-	JQ341268	AF141025	JN413119
	Calathea capitata	-	-	JQ341271	AF141026	JQ341219
	Calathea makoyana	19073768 (BR)	KU215274	KU215049	KU214921	KU215175
	Calathea majestica	19700532 (BR)	KU215276	KU215051	KU214923	KU215177
	Calathea marantifolia	19760485 (BR)	KU215270	KU215043	KU214915	KU215171
	Calathea metallica	-	-	AY673046	AF141030	AY140354
	Calathea micans	-	-	JQ341289	AF141031	JN413140
	Calathea microcephala	19700740 (BR)	KU215281	-	KU214932	-
	Calathea mirabilis	19700537 (BR)	-	KU215045	KU214917	KU215173
	Calathea petersenii	-	-	JQ341294	AF141032	JQ341237
	Calathea picturata	Van Caeckenberghe s.n. (BR)	-	KU215055	KU214928	-
	Calathea rufibarba	-	-	AY673048	AF141035	AY140360
	Calathea undulata	Van Caeckenberghe s.n. (BR)	KU215273	KU215048	KU214920	-
	Calathea variegata	19820964 (BR)	KU215272	KU215047	KU214919	-
	Calathea warscewiczii	-	-	AY673049	AY656139	AY140364
	Calathea zebrina	19700743 (BR)	-	KU215053	KU214925	-
	Ctenantha amabilis	19391944 (BR)	KU215279	KU215058	KU214930	KU215178
	Ctenantha kummerana	19073861 (BR)	KU215277	KU215056	-	-
	Ctenantha oppenheimiana	19680371 (BR)	KU215280	KU215059	KU214931	KU215179
	Ctenantha setosa	19620507 (BR)	KU215278	KU215057	KU214929	KU215190
	Donax cannaeformis	19733425 (BR)	-	KU215069	KU214945	KU215191
	Goeppertia lindeniana	19700741 (BR)	-	KU215052	KU214924	-
	Goeppertia louisea	Van Caeckenberghe s.n. (BR)	KU215271	KU215046	KU214918	KU215174
	Goeppertia nigricans	19391641 (BR)	KU215275	KU215050	KU214922	KU215176
	Goeppertia orbifolia	19910146-23 (BR)	-	KU215044	KU214916	KU215172

	Goeppertia sp.	19391639 (BR)	-	-	KU214927	-
	Halopegia azurea	-	-	AY914650	AF141048	AY140372
	Hypselodelphys hirsuta	Van Caeckenberghe s.n. (BR)	KU215288	KU215064	KU214939	KU215186
	Indianthus virgatus	-	-	AY914666	AY914620	AY140411
	Ischnosiphon heleniae	-	-	AY673055	AY656145	AY140379
	Ischnosiphon leucophaeus	-	-	JQ341309	AF141053	JN413162
	Ischnosiphon ovatus	Van Caeckenberghe s.n. (BR)	KU215291	KU215067	KU214942	-
	Maranta bicolor	19074039 (BR)	KU215283	KU215061	KU214934	KU215181
	Maranta depressa	19700747 (BR)	KU215282	KU215060	KU214933	KU215180
	Maranta leuconeura	19660030 (BR)	KU215284	KU215062	KU214935	KU215182
	Maranta lietzei	19073766 (BR)	-	KU215054	KU214926	-
	Marantochloa conferta	Van Caeckenberghe s.n. (BR)	-	-	KU214943	KU215188
	Marantochloa leucantha	19547056 (BR)	KU215287	-	KU214938	KU215185
	Marantochloa congensis	-	-	EU605903	AF141062	EU647811
	Marantochloa filipes	19850681 (BR)	-	KU215070	KU214946	KU215192
	Marantochloa purpurea	-	-	AY673057	AF141067	AY140389
	Megaphrynium macrostachyum	10005583 (BR)	-	KU215071	KU214947	KU215193
	Monotagma laxum	-	-	AY673058	AY656148	AY140392
	Phrynium giganteum	-	-	AY673050	EF382848	AY140365
	Phrynium imbricatum	-	-	AY673059	AY656149	AY140402
	Phrynium maximum	-	-	AF434901	AF430118	AY140398
	Phrynium pubinerve	-	-	JQ341264	AY914638	JQ341212
	Pleiostachya pruinosa	19910154-31 (BR)	KU215286	KU215063	KU214937	KU215184
	Sarantha sp	19575002 (BR)	KU215289	KU215065	KU214940	KU215187
	Sarcophrynium brachystachys	19910155-32 (BR)	KU215285	KU215072	KU214948	KU215194
	Sarcophrynium priogonium	10005466 (BR)	-	-	KU214936	KU215183
	Schumannianthus dichotomus	-	-	AY673064	AY656154	AY140410
	Stachyphrynium latifolium	-	-	AY914653	AY914607	AY140412
	Stachyphrynium repens	-	-	AY673060	AY656150	AY140403
	Stachyphrynium spicatum	-	-	AY914658	AY914612	AY140415
	Stachyphrynium sumatranum	-	-	AY914659	AY914613	AY140400
	Stromanthe porteana	19710269 (BR)	KU215290	KU215066	KU214941	-
	Thalia dealbata	-	-	AY914693	AY914648	AY140419
	Thalia multiflora	19770092 (BR)	KU215292	KU215068	KU214944	KU215189
	Thaumatococcus daniellii	-	-	AY673067	AF141091	AY140421
	Trachyphrynium braunianum	-	-	EU605916	AY656158	AY140377
**Musaceae**	*Ensete superbum*	-	-	FJ621291	-	FJ621291
	Ensete gilletii	ITC1389	KU215319	KU215101	KU214977	-
	Ensete glaucum	-	FJ428019	FJ428154	FJ428124	FJ428154
	Ensete glaucum	ITC0775	KU215306	KU215088	KU214963	-
	Ensete homblei	-	-	FJ621290	-	FJ621290
	Ensete ventricosum	ITC1387	-	KU215100	KU214976	KU215215
	Musa acuminata ssp. banksii	ITC0617	KU215302	KU215083	KU214959	KU215206
	Musa acuminata ssp. banksii *	ITC0619	KU215304	KU21<br>5085	-	KU215208
	Musa acuminata ssp. banksii	ITC0879	KU215307	KU215089	KU214964	-
	Musa acuminata ssp. banksii	ITC0896	KU215309	KU215090	KU214966	KU215211

Musa acuminata ssp. burmannica -	-	FJ428041	FJ428083	FJ428135	FJ428169
Musa acuminata ssp. burmannicoides -	-	FJ428044	FJ428085	FJ428133	FJ428170
*Musa acuminata ssp. errans **	-	FJ428051	FJ428094	FJ428126	FJ428160
Musa acuminata ssp. malaccensis	ITC0609	-	KU176107	KU176108	KU176109
*Musa acuminata ssp. malaccensis **	ITC1511	KU215320	KU215102	KU214978	KU215205
*Musa acuminata ssp. microcarpa **	ITC0253	KU215296	KU215076	KU214952	KU215198
Musa acuminata ssp. siamea -	ITC0672	KU215338	KU215122	KU214997	-
*Musa acuminata ssp. truncata **	ITC0393	KU215340	KU215124	KU214999	KU215218
Musa acuminata ssp. zebrina	ITC0728	-	KU215087	KU214962	-
Musa acuminata ssp. zebrina	ITC1177	KU215316	KU215097	KU214973	-
Musa acuminata ssp. zebrina	ITC1178	KU215317	KU215098	KU214974	-
*Musa acuminata ssp. zebrina **	ITC1179	KU215318	KU215099	KU214975	-
Musa aurantiaca °	-	FJ428037	FJ428090	FJ428127	FJ428162
Musa balbisiana °	ITC0247	KU215294	KU215074	KU214950	KU215196
Musa balbisiana	ITC0565	KU215300	KU215080	KU214956	KU215202
Musa balbisiana	ITC1587	KU215330	KU215114	KU214989	-
*Musa barioensis **	ITC1568	KU215328	KU215112	KU214987	-
Musa basjoo -	ITC0061	KU215293	KU215073	KU214949	KU215195
*Musa beccarii var. beccarii **	-	FJ428028	FJ428065	FJ428120	FJ428189
*Musa beccarii var. hottana **	-	FJ428029	FJ428066	FJ428115	FJ428190
*Musa borneensis **	ITC1531	KU215326	KU215110	KU214985	-
*Musa campestris ssp. sarawakensis **	ITC1517	KU215322	KU215104	KU214980	-
Musa cheesmanii °	ITC1519	KU215323	KU215106	KU214982	-
Musa coccinea °	ITC0287	KU215298	KU215078	KU214954	KU215200
Musa exotica °	ITC1532	KU215327	KU215111	KU214986	-
*Musa gracilis **	-	FJ428022	FJ428075	FJ428111	FJ428194
*Musa hirta **	-	FJ428026	FJ428074	FJ428117	FJ428199
*Musa ingens **	-	FJ428036	FJ428077	FJ428118	FJ428184
Musa itinerans ssp. xishuangbanaensis °	ITC1526	KU215325	KU215108	KU214984	-
Musa jackeyi	ITC0588	KU215301	KU215081	KU214957	KU215203
Musa laterita °	ITC1076	KU215315	KU215096	KU214972	-
*Musa lolodensis **	ITC0956	KU215313	KU215094	KU214970	KU215213
Musa lutea °	ITC1515	KU215321	KU215103	KU214979	-
*Musa maclayi ssp. ailulai **	ITC0614	KU215332	KU215116	KU214991	KU215216
*Musa maclayi ssp. maclayi var. maclayi **	ITC0864	KU215335	KU215119	KU214994	-
*Musa maclayi ssp. maclayi var. namatani **	ITC0915	KU215310	KU215091	KU214967	KU215212
Musa mannii °	ITC0543	KU215299	KU215079	KU214955	KU215201
*Musa monticola **	-	FJ428049	FJ428073	FJ428119	FJ428191
Musa nagensium °	-	FJ428058	FJ428101	FJ428144	FJ428158
Musa ornata °	ITC0637	KU215333	KU215117	KU214992	-
*Musa peekelii ssp. peekelii **	ITC0917	KU215311	KU215092	KU214968	-
*Musa peekelii sss. angustigemma **	ITC0618	KU215303	KU215084	KU214960	KU215207
Musa peekelii ssp. angustigemma	ITC0625	KU215305	KU215086	KU214961	KU215209

	Musa rosea -	-	FJ428045	FJ428080	FJ428131	FJ428171
	Musa rubinea °	-	FJ428048	FJ428093	FJ428128	FJ428163
	Musa rubra °	ITC1590	KU215331	KU215115	KU214990	-
	Musa salaccensis *	-	FJ428023	FJ428072	FJ428112	FJ428196
	Musa schizocarpa	ITC0599	-	KU215082	KU214958	KU215204
	Musa schizocarpa	ITC0890	KU215308	-	KU214965	KU215210
	Musa schizocarpa	ITC0926	KU215312	KU215093	KU214969	-
	Musa schizocarpa *	ITC1002	KU215336	KU215120	KU214995	-
	Musa siamensis -	-	FJ428047	FJ428086	FJ428134	FJ428168
	Musa textilis *	ITC1072	KU215314	KU215095	KU214971	KU215214
	Musa tonkinensis °	-	FJ428055	FJ428099	FJ428146	FJ428178
	Musa velutina °	ITC0638	KU215334	KU215118	KU214993	-
	Musa violescens *	ITC1514	KU215339	KU215123	KU214998	KU215217
	Musa viridis °	ITC1525	KU215324	KU215107	KU214983	-
	Musa yunnanensis °	ITC1573	KU215329	KU215113	KU214988	-
	Musella lasiocarpa	-	FJ428021	FJ428155	FJ428123	FJ428155
**Strelitziaceae**	*Phenakospermum guyannense*	19812682 (BR)	-	-	KU215000	KU215219
	Ravenala madagascariensis	-	FJ428017	FJ428107	FJ428110	FJ428182
	Strelitzia reginae	-	-	FJ626403	JQ027166	FJ621298
**Zingiberaceae**	*Aframomum danielii*	20030092-77 (BR)	KU215362	KU215148	KU215024	KU215239
	Aframomum hanburyi	Van Caeckenberghe s.n. (BR)	KU215361	KU215147	KU215023	-
	Aframomum corrorima	Van Caeckenberghe s.n. (BR)	KU215364	KU215150	-	KU215241
	Aframomum luteoalbum	-	-	AF414493	AF414546	FJ848664
	Aframomum thonneri	20030090-75 (BR)	KU215363	KU215149	-	KU215240
	Aframomum verrucosum	-	-	AF414492	AF414545	FJ848660
	Alpinia luteocarpa	19880195 (BR)	KU215342	KU215125	KU215001	KU215220
	Alpinia purpurata	19880109 (BR)	KU215343	KU215126	KU215002	KU215221
	Boesenbergia rotunda	20040188-85 (BR)	-	KU215139	KU215016	-
	Boesenbergia sp	19710472 (BR)	-	KU215135	KU215012	KU215229
	Cautleya lutea	20091405-86 (BR)	KU215355	KU215141	KU215018	KU215234
	Curcuma longa	19670244 (BR)	KU215347	-	KU215006	KU215224
	Curcuma zanthorrhiza	19750147 (BR)	KU215348	KU215130	KU215007	-
	Etlingera elatior	19560132 (BR)	-	-	KU215025	KU215242
	Etlingera yunnanensis	-	-	AF414468	AF414521	AY769809
	Globba schomburgkii	19741200 (BR)	KU215357	KU215143	KU215020	KU215236
	Hedychium coronarium	19870230 (BR)	-	KU215134	KU215011	KU215228
	Hedychium cylindricum	19520915 (BR)	KU215350	KU215132	KU215009	KU215226
	Kaempferia elegans	20060023-35 (BR)	KU215353	KU215138	KU215015	KU215232
	Hedychium flavescens	19800483 (BR)	KU215351	KU215133	KU215010	KU215227
	Hedychium horsfieldii	19602071 (BR)	KU215349	KU215131	KU215008	KU215225
	Kaempferia gilbertii	19570275 (BR)	KU215352	KU215136	KU215013	KU215230
	Kaempferia rotunda	19074013 (BR)	-	KU215137	KU215014	KU215231
	Kaempferia sp.	Van Caeckenberghe s.n. (BR)	KU215360	KU215146	KU215022	KU215238
	Renealmia alpina	19921107-23 (BR)	KU215358	KU215144	KU215021	-
	Renealmia nicolaioides	19750423 (BR)	KU215354	KU215140	KU215017	KU215233
	Renealmia cernua	-	-	AF414476	AF414529	DQ444517

	Riedelia sp.	19620382 (BR)	KU215359	KU215145	-	KU215237
	Siphonochilus decorus	19870078 (BR)	-	-	KU215026	KU215243
	Siphonochilus kirkii	20040213-13 (BR)	KU215356	KU215142	KU215019	KU215235
	Zingiber darceyi	Van Caeckenberghe s.n. (BR)	KU215346	KU215129	KU215005	-
	Zingiber officinale	19920009-89 (BR)	-	-	KU215027	KU215244
	Zingiber papuanum	19763771 (BR)	KU215344	KU215127	KU215003	KU215222
	Zingiber zerumbet	19520932 (BR)	KU215345	KU215128	KU215004	KU215223
**Typhaceae**	*Sparganium sp.*	-	JF280745	-	HQ913892	HQ882765
	Typha angustifolia	-	FJ914237	-	AM116858	JF319450

Table S2 Dispersal probability between the different Southeast Asian (and African) areas throughout four defined time periods (0–10 Mya (Pleistocene to Late Miocene), 10–20 Mya (Late to Early Miocene), 20–30 Mya (Early Miocene to Oligocene) and 30–55 Mya (Oligocene to Early Eocene) used as input for the ancestral area analyses conducted by Lagrange. Due to the complex evolution of the Southeast Asian subcontinent, dispersal probabilities (DP) differ in time (DP 1.0: no dispersal restriction between Southeast Asian distribution ranges, DP 0.5: limited dispersal as certain landmasses (e.g. New Guinea) were not fully emerged yet, DP 0.0: Land mass not yet existing during defined time period. A, Africa; B, Southwest India and Sri Lanka; C, northern Indo-Burma; D, South China; E, southern Indo-Burma; F, Sumatra and Malayan Peninsula; G, Borneo; H, Philippines; I, New Guinea and surrounding islands; J, Northwest Australia; K, Lesser Sunda Islands; L, Sulawesi.

0 to 10	A	B	C	D	E	F	G	H	I	J	K	L
A	--	1.0	1.0	1.0	1.0	1.0	1.0	1.0	1.0	1.0	1.0	1.0
B	1.0	--	1.0	1.0	1.0	1.0	1.0	1.0	1.0	1.0	1.0	1.0
C	1.0	1.0	--	1.0	1.0	1.0	1.0	1.0	1.0	1.0	1.0	1.0
D	1.0	1.0	1.0	--	1.0	1.0	1.0	1.0	1.0	1.0	1.0	1.0
E	1.0	1.0	1.0	1.0	--	1.0	1.0	1.0	1.0	1.0	1.0	1.0
F	1.0	1.0	1.0	1.0	1.0	--	1.0	1.0	1.0	1.0	1.0	1.0
G	1.0	1.0	1.0	1.0	1.0	1.0	--	1.0	1.0	1.0	1.0	1.0
H	1.0	1.0	1.0	1.0	1.0	1.0	1.0	--	1.0	1.0	1.0	1.0
I	1.0	1.0	1.0	1.0	1.0	1.0	1.0	1.0	--	1.0	1.0	1.0
J	1.0	1.0	1.0	1.0	1.0	1.0	1.0	1.0	1.0	--	1.0	1.0
K	1.0	1.0	1.0	1.0	1.0	1.0	1.0	1.0	1.0	1.0	--	1.0
L	1.0	1.0	1.0	1.0	1.0	1.0	1.0	1.0	1.0	1.0	1.0	--

10 to 20	A	B	C	D	E	F	G	H	I	J	K	L
A	--	1.0	1.0	1.0	1.0	1.0	1.0	1.0	0.5	1.0	0.5	1.0
B	1.0	--	1.0	1.0	1.0	1.0	1.0	1.0	0.5	1.0	0.5	1.0
C	1.0	1.0	--	1.0	1.0	1.0	1.0	1.0	0.5	1.0	0.5	1.0
D	1.0	1.0	1.0	--	1.0	1.0	1.0	1.0	0.5	1.0	0.5	1.0
E	1.0	1.0	1.0	1.0	--	1.0	1.0	1.0	0.5	1.0	0.5	1.0
F	1.0	1.0	1.0	1.0	1.0	--	1.0	1.0	0.5	1.0	0.5	1.0
G	1.0	1.0	1.0	1.0	1.0	1.0	--	1.0	0.5	1.0	0.5	1.0
H	1.0	1.0	1.0	1.0	1.0	1.0	1.0	--	0.5	1.0	0.5	1.0
I	0.5	0.5	0.5	0.5	0.5	0.5	0.5	0.5	--	0.5	0.5	0.5
J	1.0	1.0	1.0	1.0	1.0	1.0	1.0	1.0	0.5	--	0.5	1.0
K	0.5	0.5	0.5	0.5	0.5	0.5	0.5	0.5	0.5	0.5	--	1.0
L	1.0	1.0	1.0	1.0	1.0	1.0	1.0	1.0	0.5	1.0	1.0	--

20 to 30	A	B	C	D	E	F	G	H	I	J	K	L
A	--	1.0	1.0	1.0	1.0	1.0	1.0	0.5	0.5	1.0	0.5	1.0
B	1.0	--	1.0	1.0	1.0	1.0	1.0	0.5	0.5	1.0	0.5	1.0
C	1.0	1.0	--	1.0	1.0	1.0	1.0	0.5	0.5	1.0	0.5	1.0
D	1.0	1.0	1.0	--	1.0	1.0	1.0	0.5	0.5	1.0	0.5	1.0
E	1.0	1.0	1.0	1.0	--	1.0	1.0	0.5	0.5	1.0	0.5	1.0
F	1.0	1.0	1.0	1.0	1.0	--	1.0	0.5	0.5	1.0	0.5	1.0
G	1.0	1.0	1.0	1.0	1.0	1.0	--	0.5	0.5	1.0	0.5	1.0
H	0.5	0.5	0.5	0.5	0.5	0.5	0.5	--	0.5	1.0	0.5	1.0
I	0.5	0.5	0.5	0.5	0.5	0.5	0.5	0.5	--	0.5	0.5	0.5
J	1.0	1.0	1.0	1.0	1.0	1.0	1.0	1.0	0.5	--	0.5	1.0
K	0.5	0.5	0.5	0.5	0.5	0.5	0.5	0.5	0.5	0.5	--	1.0
L	1.0	1.0	1.0	1.0	1.0	1.0	1.0	1.0	0.5	1.0	1.0	--

30 to 55	A	B	C	D	E	F	G	H	I	J	K	L
A	--	1.0	1.0	1.0	1.0	1.0	1.0	0.0	0.0	0.0	0.0	0.0
B	1.0	--	1.0	1.0	1.0	1.0	1.0	0.0	0.0	0.5	0.0	0.0
C	1.0	1.0	--	1.0	1.0	1.0	1.0	0.0	0.0	0.5	0.0	0.0
D	1.0	1.0	1.0	--	1.0	1.0	1.0	0.0	0.0	0.5	0.0	0.0
E	1.0	1.0	1.0	1.0	--	1.0	1.0	0.0	0.0	0.5	0.0	0.0
F	1.0	1.0	1.0	1.0	1.0	--	1.0	0.0	0.0	0.5	0.0	0.0
G	1.0	1.0	1.0	1.0	1.0	1.0	--	0.0	0.0	0.5	0.0	0.0
H	0.0	0.0	0.0	0.0	0.0	0.0	0.0	--	0.0	0.0	0.0	0.0
I	0.0	0.0	0.0	0.0	0.0	0.0	0.0	0.0	--	0.5	0.0	0.0
J	0.0	0.5	0.5	0.5	0.5	0.5	0.5	0.0	0.5	--	0.0	0.0
K	0.0	0.0	0.0	0.0	0.0	0.0	0.0	0.0	0.0	0.0	--	0.0
L	0.0	0.0	0.0	0.0	0.0	0.0	0.0	0.0	0.0	0.0	0.0	--

Table S3 Sequence characteristics of ITS, *atpB-rbcL*, *trnL-F* and *rps16*, and the combined matrix

	ITS	*atpB-rbcL*	*trnL-F*	*rps16*	combined
Analyzed characters	1908	1086	2060	1750	6807
Variable characters	807	211	467	494	1979
PI characters	612	126	278	147	1163
No. of taxa	201	142	180	213	227

PI, parsimony informative

Table S4. Model fit of area-dependent diversification. Eight models with diverse constraints and degrees of freedom (df) were selected for Bayesian model averaging (BMA). Subscript 0 refers to an Indo-Burmese distribution, whereas subscript 1 refers a Malesian distribution. Bayes factors testing support the selected model of equal speciation rates, equal transition rates and a null extinction for species occurring in Indo-Burma.

BiSSE models											
**λ_0**		**λ_1**	**μ_0**		**μ_1**	**q_{01}**		**q_{10}**	**df**	**LogMarg**	**BF**
λ_0	=	λ_1	0			q_{01}	=	q_{10}	3	-183.83	0.0
λ_0	=	λ_1			0	q_{01}	=	q_{10}	3	-185.27	2.88
λ_0	=	λ_1				q_{01}	=	q_{10}	4	-185.45	3.24
			0		0	q_{01}	=	q_{10}	2	-190.34	13.02
						q_{01}	=	q_{10}	5	-190.82	13.98
			0			q_{01}	=	q_{10}	4	-191.30	14.94
					0	q_{01}	=	q_{10}	4	-191.61	15.56
			μ_0	=	μ_1	q_{01}	=	q_{10}	4	-192.25	16.84

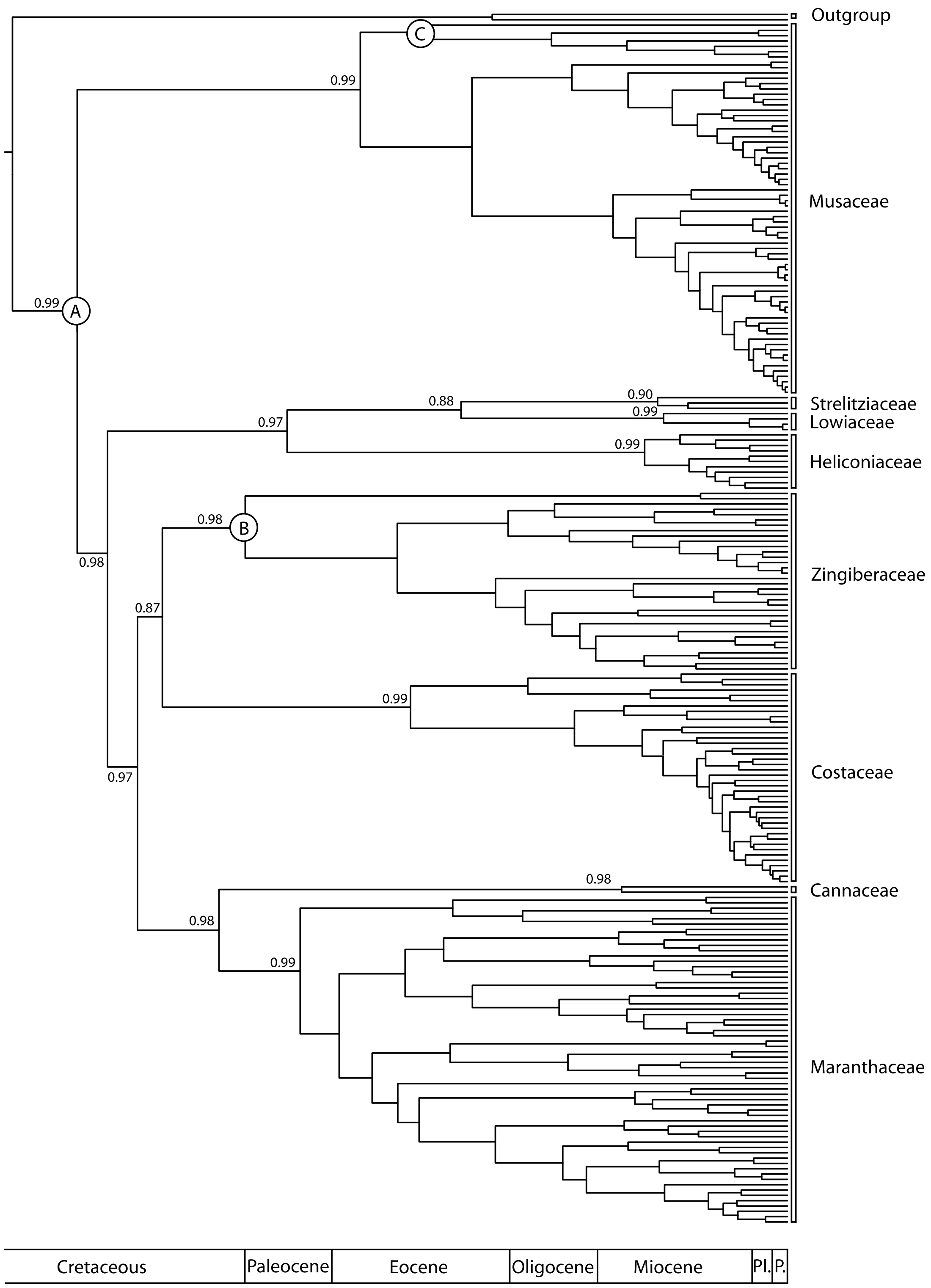

Fig. S1 BEAST Maximum clade credibility tree of Zingiberales inferred from combined ITS, *trnL-F*, *rps16* and *atpB-rbcl*. Bayesian Posterior Probabilities at family level or higher are added above the branches. Calibration points are indicated at selected nodes: (A) secondary calibration point Zingiberales, (B) *Zingiberopsis attenuata* and (C) *Ensete oregonense*. Pl., Pliocene; P., Pleistocene.

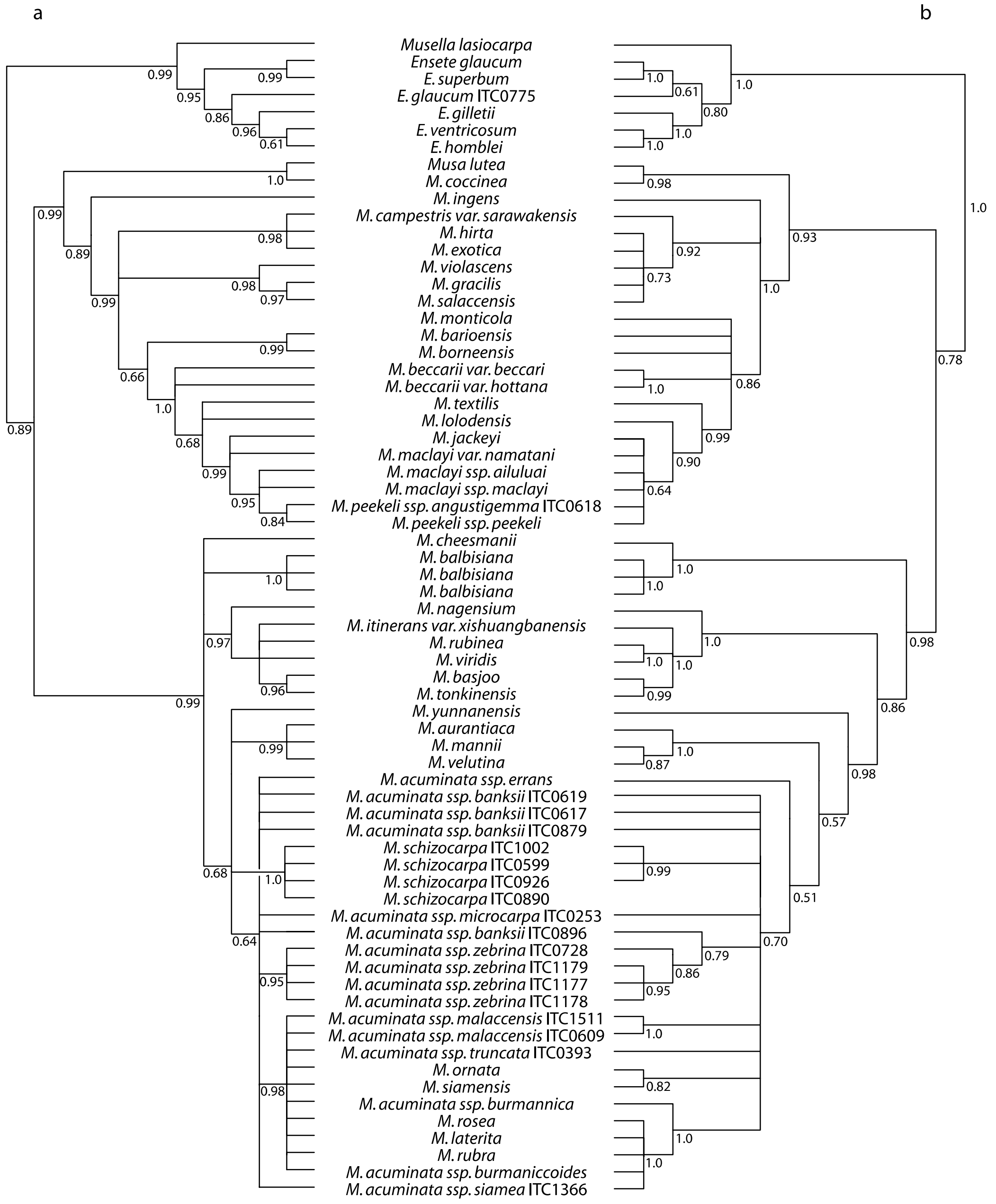

Fig. S2 Bayesian consensus phylogram of (a) the chloroplast (*trnL-F*, *rps16* and *atpB-rbcL*) dataset and (b) nuclear (ITS) dataset. Bayesian Posterior probability values are indicated above or below the branches.

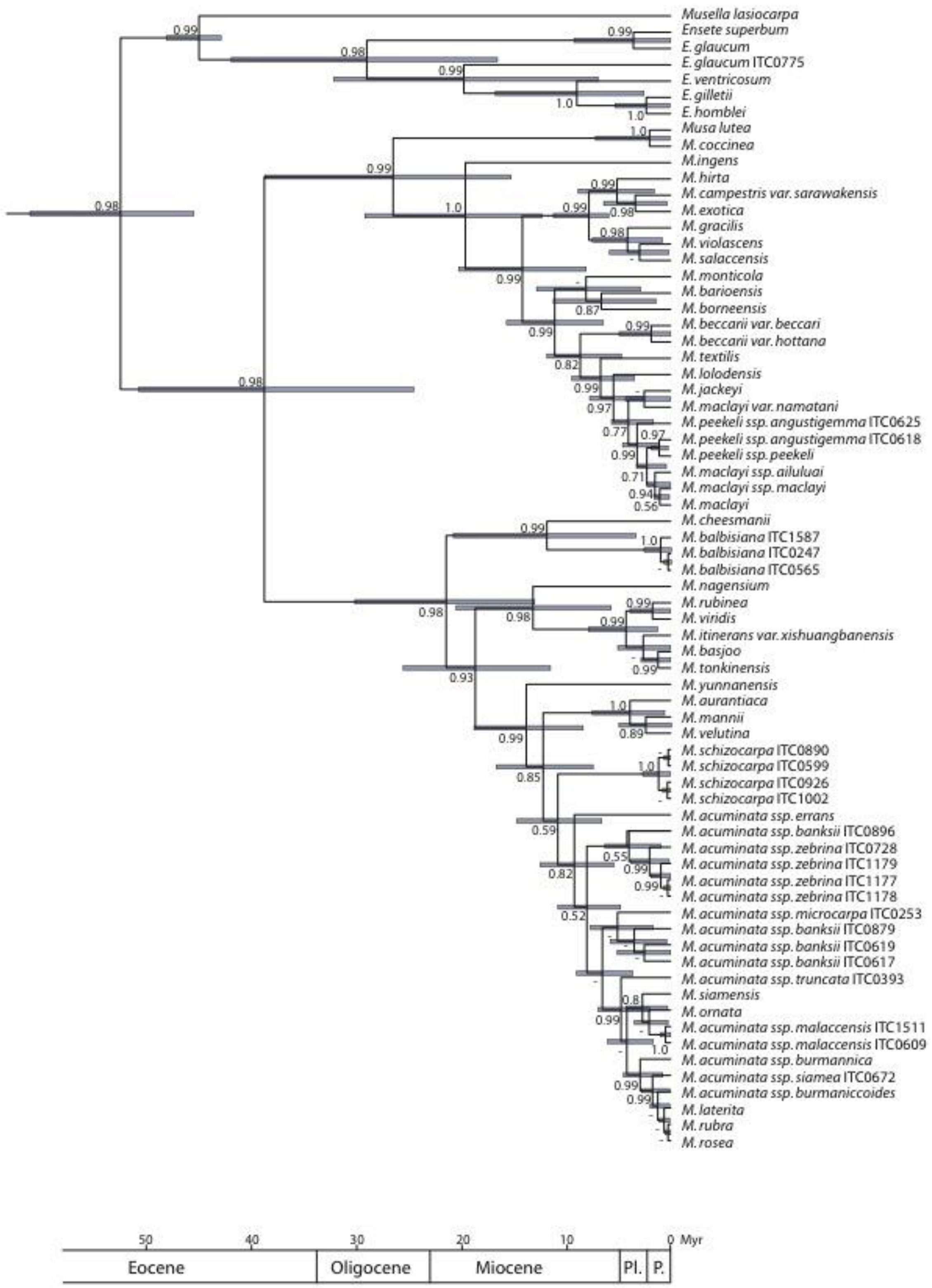

Fig. S3 Maximum clade credibility tree of the combined ITS, *atpB-rbcL*, *rps16* and *trnL–trnF* dataset of the Musaceae from the BEAST analysis (detail of the large Zingiberales phylogram of Fig. S1). Blue bars indicate age intervals (95% HPD credibility). Bayesian Posterior probability values are indicated above or below the branches. Unsupported nodes present in the maximum clade credibility tree are indicated by a hyphen.

Methods S1 Taxon sampling.

The taxon sampling of this study was chosen to represent the geographic and taxonomic diversity of the Musaceae family. In total 5 *Ensete* species (6 accessions), 38 *Musa* species (63 accessions) and 1 species (1 accession) of *Musella* were included. Furthermore, in order to correctly estimate node ages for *Ensete*, *Musa* and *Musella*, we extended the Musaceae dataset – which is the focal point of this study – with a substantial number of genera and species of all known Zingiberales families: Heliconiaceae (1 genus, 11 species), Lowiaceae (1 genus, 4 species), Strelitziaceae (3 genera, 3 species), Zingiberaceae (13 genera, 34 species), Marantaceae (23 genera, 62 species), Cannaceae (1 genus, 2 species) and Costaceae (6 genera, 40 species). Within the Zingiberales dataset, each species was represented by one accession. Newly obtained sequences were obtained from fresh or silica-dried leaf material. For the Musaceae accessions, we obtained *in vitro* rooted plants of most of the wild *Musa* and *Ensete* species from the International Transit Centre, Bioversity International (based at KU Leuven, Belgium), whereas extant Zingiberales samples were retrieved from the living plant collection of the Botanic Garden Meise (formerly known as National Botanic Garden Belgium). *Typha angustifolia* and *Sparganium* sp. (Typhaceae) were selected as outgroup species to the large Zingiberales dataset. Information about species names, voucher information and GenBank accessions are provided in Table S1.

Methods S2 Molecular protocols and sequence analyses.

A modified CTAB protocol was used for total genomic DNA isolation (Tel-Zur *et al.*, 1999). Amplification reactions of *rps16*, *atpB-rbcL, trnL-F* and ITS were carried out following primers and protocols of Oxelman *et al.* (1997), Chiang *et al.* (1998), Taberlet *et al.* (1991) and White *et al.* (1990), respectively. Purified amplification products were sequenced by the Macrogen sequencing facilities (Macrogen Europe, Amsterdam, Netherlands). Raw sequences were assembled using Geneious v7.0.6 (Biomatters, Auckland, New Zealand). Automatic alignment was conducted with MAFFT (Katoh *et al.*, 2002) using an E-INS-i algorithm, a 100PAM/k=2 scoring matrix, a gap open penalty of 1.3 and an offset value of 0.123. Manual fine-tuning of the aligned dataset was performed in Geneious v7.0.6.

Congruency between the different datasets was inferred using different methods. First, a series of partition homogeneity test (Farris *et al.*, 1995) were carried out with PAUP* 4.0b10 (Swofford, 2003). Pairwise tests were performed between datasets of different genomic origin (combined plastid dataset and nuclear ribosomal ITS dataset). Despite the well-known sensitivity of the partition homogeneity test (Barker & Lutzoni, 2002), the results of this test were compared in light of the resolution and support values of the obtained plastid and nuclear topologies. As a result, possible conflict between data matrices was visually inspected, searching for conflicting relationships within each topology that are strongly supported (hard vs. soft incongruence; Johnson & Soltis, 1998).

The best-fit nucleotide substitution model for each plastid and nuclear dataset was selected using jModelTest 2.1.4. (Posada, 2008) under the Akaike information criterion (AIC). The GTR+I+G model was determined as best fit for *rps16* and *trnL-F*, GTR+G for *atpB-rbcL* and F81+I for ITS. Likewise, we used a mixed-model approach to apply different evolutionary models on each DNA region of the combined dataset (Ronquist & Huelsenbeck, 2003). Bayesian inference analyses were conducted with MrBayes 3.1 (Huelsenbeck & Ronquist, 2001) on four individual data partitions and a combined data matrix. Each analysis was run two times for 20 million generations. Trees were sampled every 5,000th generation. Chain convergence and ESS parameters were inspected with TRACER 1.4 (Rambaut & Drummond, 2007). Only nodes with Bayesian posterior probabilities (BPP) above 0.95 are considered as well supported (Suzuki *et al.*, 2002).

Methods S3 BEAST detail methods, parameters and settings.

BEAST 1.8.0 (Drummond & Rambaut, 2007) was used to compute divergence times. However, in order to surpass the zero likelihood issue in BEAST, we used a starting tree that was obtained by carrying out a ML analysis in RAxML 7.2.8 (Stamatakis *et al.*, 2008) under GTRGAMMA model with the rooted likelihood tree as input tree for a penalized likelihood (PL) analysis in the software program PL-tree (Wang *et al.*, 2013). PL-tree is based on the Penalized Likelihood algorithm of Sanderson (2002). All calibration points are used as described above. Due to differing substitution models for the plastid and nuclear gene markers, a partitioned Bayesian MCMC analysis was performed under the Yule speciation model and a relaxed lognormal clock. Partitions were unlinked for the model of evolution. The analysis ran for 30 million generations and was sampled each 5,000th generation. Convergence of the chains and ESS parameter evaluation (ESS > 200) was performed with TRACER 1.6 (Rambaut & Drummond, 2007). A maximum clade credibility tree using a posterior probability limit of 0.5 was calculated using TreeAnnotator 1.8.0. (Drummond & Rambaut, 2007).

References

Barker FK, Lutzoni FM. 2002. The utility of the incongruence length difference test. *Systematic Biology* **51**: 625–637.

Chiang TY, Schaal BA, Peng C-I. 1998. Universal primers for amplification and sequencing a non-coding spacer between the *atpB* and *rbcL* genes of chloroplast DNA. *Botanical Bulletin of Academia Sinica* **39**: 245–250.

Drummond AJ, Rambaut A. 2007. BEAST: Bayesian evolutionary analysis by sampling trees. *BMC Biology* **7**: 214.

Farris JD, Källersjö M, Kluge AG, Bult C. 1995. Constructing a significance test for incongruence. *Systematic Biology* **44**: 570–572.

Huelsenbeck JP, Ronquist F. 2001. MRBAYES, Bayesian inference of phylogenetic trees. *Bioinformatics* **17**: 754–755.

Johnson LA, Soltis DE. 1998. Assessing congruence: Empirical examples from molecular data. In: Soltis PS, Soltis DE, Doyle JJ, eds. *Molecular Systematics of Plants II: DNA Sequencing*. Boston, MA, USA: Kluwer, 297-348.

Katoh K, Misawa K, Kuma K, Miyata T. 2002. MAFFT: a novel method for rapid multiple sequence alignment based on fast Fourier transform. *Nucleic Acids Research* **30**: 3059–3066.

Oxelman B, Liden M, Berglund D. 1997. Chloroplast *rps16* intron phylogeny of the tribe Sileneae (Caryophyllaceae). *Plant Systematics and Evolution* **206**: 393–410.

Posada D. 2008. jModelTest: phylogenetic model averaging. *Molecular Biology and Evolution* **25**: 1253–1256.

Rambaut A, Drummond AJ. 2007. *Tracer v1.4*. URL http://beast.bio.ed.ac.uk/Tracer.

Ronquist F, Huelsenbeck JP. 2003. Mr. Bayes 3: Bayesian phylogenetic inference under mixed models. *Bioinformatics* **19**: 1572–1574.

Sanderson MJ. 2002. Estimating absolute rates of molecular evolution and divergence times: a penalized likelihood approach. *Molecular Biology and Evolution* **19**: 101–109.

Stamatakis A, Hoover P, Rougement J. 2008. A fast bootstrapping algorithm for the RAxML web servers. *Systematic Biology* **57**: 758–771.

Suzuki Y, Glazko GV, Nei M. 2002. Over credibility of molecular phylogenies obtained by Bayesian phylogenetics. *Proceedings of the National Academy of Sciences, USA* **99**: 16138–16143.

Swofford DL. 2003. *PAUP*: Phylogenetic Analysis Using Parsimony (*and other methods), version 4.0b10*. Sunderland, MA, USA: Sinauer Associates.

Taberlet P, Gielly L, Pautou G, Bouvet J. 1991. Universal primers for the amplification of three non-coding regions of chloroplast DNA. *Plant Molecular Biology* **17**: 1105–1109.

Tel-Zur N, Abbo S, Myslabodski D, Mizrahi Y. 1999. Modified CTAB procedure for DNA isolation from epiphytic cacti of the genera *Hylocereus* and *Selenicereus* (Cactaceae). *Plant Molecular Biology Reporter* **17**: 249–254.

Wang J, Lu J, Fang Z, Ge T, Chen C. 2013. PL-Tree: An Efficient Indexing Method for High-Dimensional Data. In: Nascimento M, Sellis T, Cheng R, Sander J, Zheng Y, Kriegel H-P, Renz M, Sengstock C, eds. *Advances in Spatial and Temporal Databases, Lecture Notes in Computer Science.* Heidelberg, Geramany: Springer, 183–200.

White TJ, Bruns TD, Lee SB, Taylor JW. 1990. Amplification and direct sequencing of fungal ribosomal RNA genes for phylogenetics. In: Innis MA, Gelfand DH, Sninsky JI, White TJ, eds. *PCR Protocols: A Guide to Methods and Applications.* San Diego, CA, USA: Academic Press, 315–322.
